# *N*-Oxide Coordination to Mn(III) Chloride

**DOI:** 10.3390/molecules29194670

**Published:** 2024-10-01

**Authors:** Ananya Saju, Matthew R. Crawley, Samantha N. MacMillan, Pierre Le Magueres, Mark Del Campo, David C. Lacy

**Affiliations:** 1 Department of Chemistry, University at Buffalo, State University of New York, Buffalo, NY 14260, USA; 2 Department of Chemistry and Chemical Biology, Cornell University, Ithaca, NY 14853, USA; 3 Rigaku Americas, The Woodlands, TX 77381, USAmark.delcampo@rigaku.com (M.D.C.)

**Keywords:** Mn(III), *N*-oxide ligands, coordination chemistry, C–H chlorination

## Abstract

We report on the synthesis and characterization of Mn(III) chloride (Mn^III^Cl_3_) complexes coordinated with *N*-oxide ylide ligands, namely trimethyl-*N*-oxide (Me_3_NO) and pyridine-*N*-oxide (PyNO). The compounds are reactive and, while isolable in the solid-state at room temperature, readily decompose into Mn(II). For example, “[Mn^III^Cl_3_(ONMe_3_)_n_]” decomposes into the 2D polymeric network compound complex salt [Mn^II^(µ-Cl)_3_Mn^II^(µ-ONMe_3_)]_n_[Mn^II^(µ-Cl)_3_]_n_·(Me_3_NO·HCl)_3n_ (**4**). The reaction of Mn^III^Cl_3_ with PyNO forms varied Mn(III) compounds with PyNO coordination and these react with hexamethylbenzene (HMB) to form the chlorinated organic product 1-cloromethyl-2,3,4,5,6-pentamethylbenzene (**8**). In contrast to *N*-oxide coordination to Mn(III), the reaction between [Mn^III^Cl_3_(OPPh_3_)_2_] and 2,2,6,6-tetramethyl-1-piperidinyloxy (TEMPO) resulted in electron transfer-forming *d*^5^ manganate of the [TEMPO] cation instead of TEMPO–Mn(III) adducts. The reactivity affected by *N*-oxide coordination is discussed through comparisons with other L–Mn^III^Cl_3_ complexes within the context of reduction potential.

## 1. Introduction

The manganese (III) ion is important in environmental and biological processes [1] and is used for various applications in organic synthesis [2,3,4,5,6,7,8,9,10,11,12]. Its importance stems from its potent reactive properties, but as a result, much of the chemistry surrounding Mn(III) halides is limited due to the lack of stable molecular precursors. For instance, Mn^III^Cl_3_ is typically prepared by treating manganese oxides with ethereal hydrochloric acid generating deep purple–brown solutions whose contents have been demonstrated to contain solvated forms of Mn^III^Cl_3_ [13,14,15,16]. These solutions are unstable above temperatures of −35 °C and are sensitive to reaction conditions. A room temperature (r.t.) meta-stable solution containing MnCl_3_ was prepared by Christou and coworkers using **Mn_12_** ([Mn_12_O_l2_(OAc)_16_(H_2_O)_4_]·2HOAc·4H_2_O) as the starting material [17]. We used “Christou’s solution” of MnCl_3_ to prepare the complex [Mn^III^Cl_3_(OPPh_3_)_2_] (**1**), which is bench stable and can be stored indefinitely as a solid open to air [18]. We hypothesized that the pnictogen-oxide ylide ligand triphenylphosphine oxide (Ph_3_PO) is responsible for the stabilization of solvated Mn^III^Cl_3_ complexes. As an extension of this hypothesis, we have begun to explore other pnictogen-oxide ylide ligands to determine how differences affect the chemical properties of Mn^III^Cl_3_. 

**Scheme 1:**
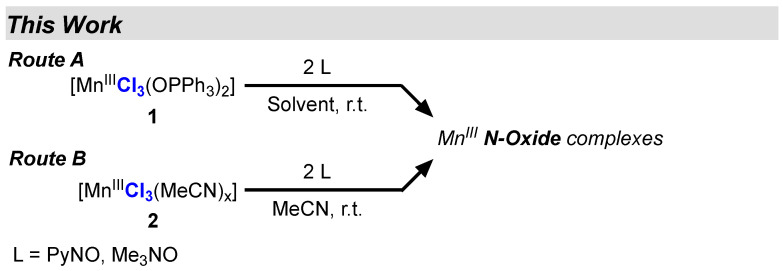
Synthesis of Mn(III) chloride complexes.

For this study, we chose to explore the synthesis of Mn(III) chloride complexes with the *N*-oxide ylide ligands pyridine-*N*-oxide (PyNO), trimethylamine-*N*-oxide (Me_3_NO), and 2,2,6,6-tetramethyl-1-piperidinyloxy (TEMPO). Me_3_NO has rarely been used as a ligand for metal complexes (ConQuest (CSD version 5.45) search on 07/24/2024 for transition metal complexes with Me_3_NO ligands produces 18 examples of the following metal ions—Re^+1^, Mn^1+^, Fe^3+^, Co^2+^, Ni^2+^, Cu^2+^, Zn^2+^, and Y^3+^), with only one example for Mn previously reported by us [19]. A few Mn(III) chloride complexes with PyNO ligand have been reported [20,21]. However, their syntheses have involved the noted cumbersome low temperature procedures, and they have not been characterized with crystallography, nor have their chemical properties been studied. We recently reported the first example of a structurally characterized PyNO ligation to Mn(III) [22]. Therefore, in this current study, we continue the coordination chemistry of *N*-oxide ligands to Mn(III) chloride complexes (Scheme 1).

## 2. Results and Discussion

### 2.1. Description of Starting Materials

Our initial success with preparing Mn(III) complexes utilized Mn^III^Cl_3_(MeCN)_x_ (x = 2, 3) generated in situ (**2**), prepared from treating **Mn_12_** with an excess of Me_3_SiCl in MeCN [18]; we refer to this solution as “Christou’s solution” and it contains **2**, Me_3_Si-derived byproducts, water, and acetic acid. While Christou’s solution is convenient, it has a non-precise stoichiometry and the noted byproducts may interfere with the desired chemistry. Furthermore, **2** is not a thermally stable compound and needs to be used soon after generation (within an hour after generation at r.t. or one day if stored at −35 °C). All our attempts at the isolation of **2** resulted in the isolation of the known molecular compound [Mn^II^Cl_2_(MeCN)_2_] [23].

We initially used **2** to prepare **1**, but we also developed a single-pot synthesis [18]. As a material, **1** is a convenient Mn^III^Cl_3_ source as it is an air-stable solid, can be weighed out accurately, and can be used in a variety of solvents such as benzene, toluene, DCM, MeCN, and acetone [18,24]. As such, **1** is the ideal starting material in most cases, but **2** can be used when Ph_3_PO interferes with the desired coordination chemistry or purification. 

### 2.2. Synthesis of Mn(III) Trimethylamine-N-Oxide Compounds

**Scheme 2:**
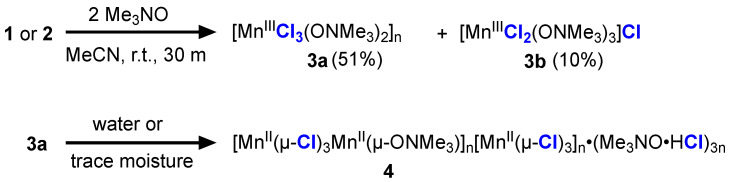
Synthesis of **3a**, **3b**, and **4**.

The reaction of Me_3_NO with **1** or **2** in MeCN produces an insoluble purple solid as the major product which we tentatively assign as [Mn^III^Cl_3_(Me_3_NO)_2_]_n_ (**3a**) for reasons that are discussed soon (Scheme 2). The FTIR spectrum of the solid shows a shifted N−O stretch of Me_3_NO at 1234 cm^−1^; free Me_3_NO has a stretch at 1254 cm^−1^. CHN combustion analysis agrees with a composition of Mn_3_Cl_9_(Me_3_NO)_5.7_. 

From the same reaction mixture, the purple solvated complex salt [Mn^III^Cl_2_(ONMe_3_)_3_]Cl·MeCN (**3b**) also forms as a minor product. The XRD analysis of **3b** revealed an Mn(III) center in pseudo trigonal bipyramidal geometry (τ_5_ = 0.52) with *trans*-Me_3_NO ligation. The FTIR spectra of (**3b**) shows an N–O stretch at 1234 cm^−1^ identical to **3a** and a C−N stretch at 2251 cm^−1^ arising from the free acetonitrile solvate. Suspensions of **3a** in MeCN with or without Me_3_NO did not increase yields of **3b**. Considering the low yield and extreme sensitivity to air and moisture and room temperature instability, **3b** was not included in the subsequent reactivity studies. 

**Figure 1:**
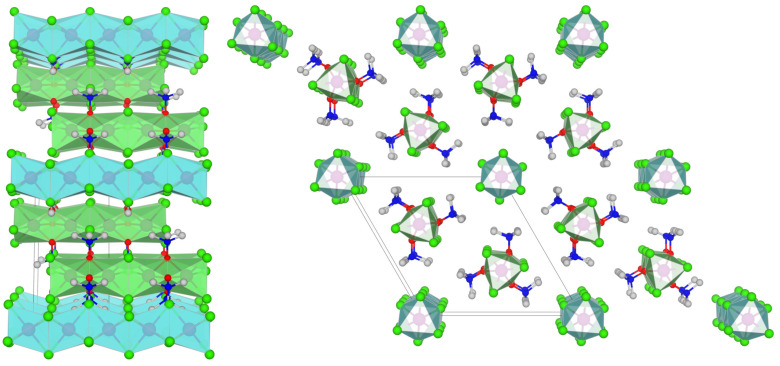
MicroED structure of [Mn^II^(µ-Cl)_3_Mn^II^(µ-ONMe_3_)]_n_[Mn^II^(µ-Cl)_3_]_n_·(Me_3_NO·HCl)_3n_ (**4**). The grey box contains the unit cell and is viewed down the b-axis (**left**) and c-axis (**right**). Selected bond lengths (Å) for the cationic chain: Mn1–Cl1 = 2.56(3); Mn1–O1 = 2.19(3); N1–O1 = 1.397(19); Mn1(µ-Cl)···Mn1(µ-O)···Mn1 = 3.27, 3.11. Selected bond lengths (Å) for the anionic chain: Mn2–Cl2 = 2.54(3); Mn2···Mn2 = 3.19. Color scheme: cyan polyhedra = [Mn^II^Cl_3_]^−^; green polyhedra = [Mn^II^_2_Cl_3_(ONMe_3_)]^+^; green sphere = Cl; magenta spheres = Mn; blue spheres = N; red spheres = O; grey spheres = C.

**Figure 2:**
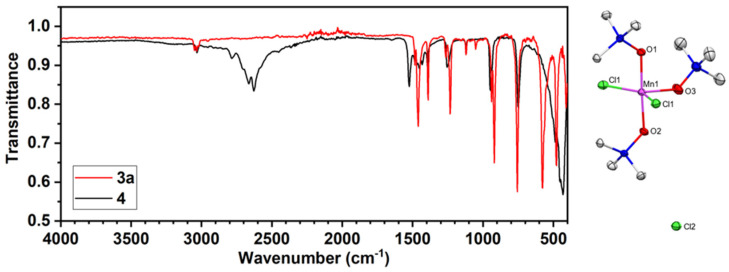
(**Left**) FTIR spectra of **3a** (red) and **4** (black). (**Right**) Molecular structure of **3b** with the outer sphere Cl counter anion shown (hydrogen atoms and MeCN are omitted for clarity). Selected bond lengths (Å) and angles (deg.) for **3a**: Mn1–Cl1 = 2.3225(6); Mn1–O1 = 1.901(2); Mn1–O2 = 1.896(2); Mn1–O3 = 1.991(3); Cl1–Mn1–Cl1 = 145.87(4); O1–Mn1–O2 = 177.33(11).

The complex **3a** is extremely reactive to moisture and immediately changes into an insoluble brown solid, **4**; this conversion occurs quickly in air (≤1 min) and even occurs in a glovebox with 1 ppm water (≤1 week). We were successful at obtaining a molecular structure of **4** using microcrystal electron diffraction (MicroED) [25,26] and this revealed that **4** is an ion pair of two manganese chloride polymers (Figure 1). The cationic polymeric species contains a repeating unit of [Mn^II^(µ-Cl)_3_Mn^II^(µ-ONMe_3_)_3_]^+^, and the anionic polymeric species has the repeating unit of [Mn^II^Cl_3_]^−^. The material from which the MicroED structure of **4** was obtained also contained crystals of Me_3_NO·HCl consistent with the characteristic broad H–Cl stretch at 2650 cm^−1^ in the FTIR spectrum (Figure 2). These data, in addition to a CHN analysis, enabled us to characterize the brown solid **4** as [Mn^II^(µ-Cl)_3_Mn^II^(µ-ONMe_3_)]_n_[Mn^II^(µ-Cl)_3_]_n_·(Me_3_NO·HCl)_3n_ (**4**). The polymeric [{Mn^II^Cl_3_}^−^]_n_ entity has been observed before [27,28,29,30] and has Mn–Cl bond lengths of 2.5–2.6 Å, in agreement with the Mn–Cl bond length for the cation (2.6 Å) and anion (2.5 Å) polymer chains in **4**, supporting our assignment of the Mn(II) oxidation states in both. Although not pursued in this study due to limited access to the MicroED instrumentation, we assumed a similar structure for the insoluble **3a**, which has intriguing similarities to certain polymers proposed in the literature to have unique electronic properties [31,32,33]; the structural elucidation of **3a** is being pursued in a separate study.

The instability of **3a** and **3b** towards the reduction to Mn(II) species is characteristic of Mn(III) complexes in general. We have shown that one path for decomposition is the reduction of Mn(III)X species by C–H bonds [22,34] and we test this hypothesis later with hexamethylbenzene (HMB) (vide infra). Two alternative paths are water oxidation or disproportionation. No gas evolution was observed, and we do not see evidence for H_2_O_2_ in the FTIR spectrum or in the structure of **4**, indicating that water oxidation is unlikely to be the decomposition pathway, albeit rigorous gas evolution studies were not performed. Disproportionation is unlikely too because we did not observe any Mn(IV) species. The structure of **4** rules out pathways involving O-atom transfers from Me_3_NO. Therefore, in the absence of an external substrate, we presume that the organic solvent or the methyl groups in Me_3_NO are acting as the reductant for **3a**. Its decomposition onset by inclusion of water is not well understood. 

### 2.3. Synthesis of Mn(III) PyNO Compounds

Two Mn(III) chloride complexes with PyNO ligands, namely [Mn^III^Cl_3_(PyNO)_2_] and [Mn^III^Cl_3_(PyNO)_3_], have been reported previously [20,21]. The synthesis of these complexes uses ethereal solutions of Mn^III^Cl_3_ that require manipulations below −35 °C and the products of these reactions are only partially characterized. Furthermore, there are some dubious aspects of the assignments that likely arise from the difficulty in assigning the empirical formulas to paramagnetic compounds from CHN analysis alone. 

**Scheme 3:**
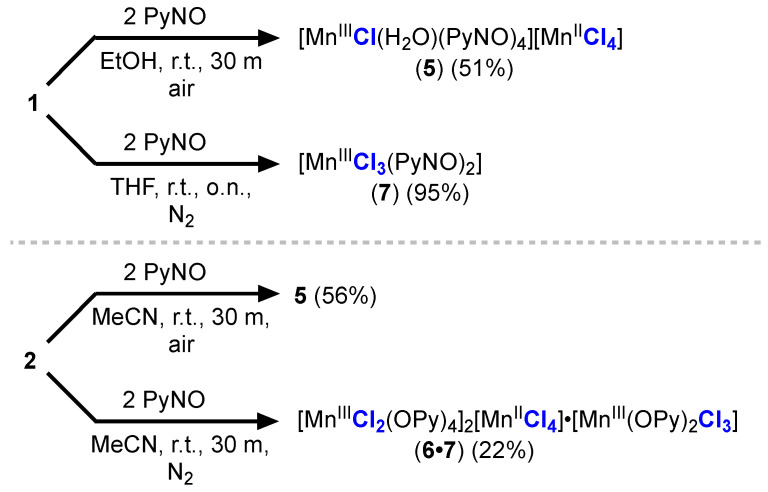
Synthesis of **5**, **6**, and **7**. Small changes in reaction conditions give different products.

Following a similar procedure from Uson to prepare [Mn^III^Cl_3_(PyNO)_2_] [20], we reacted PyNO with **1** in EtOH. Rather than forming [Mn^III^Cl_3_(PyNO)_2_] as noted in the literature [20], we obtained the complex salt [Mn^III^Cl(H_2_O)(PyNO)_4_][Mn^II^Cl_4_] (**5**) (Scheme 3) and it was characterized by X-ray diffraction (Figure 2); the same product was formed when PyNO was reacted with Christou’s solution, i.e., **2** in MeCN, open to air. 

**Figure 3:**
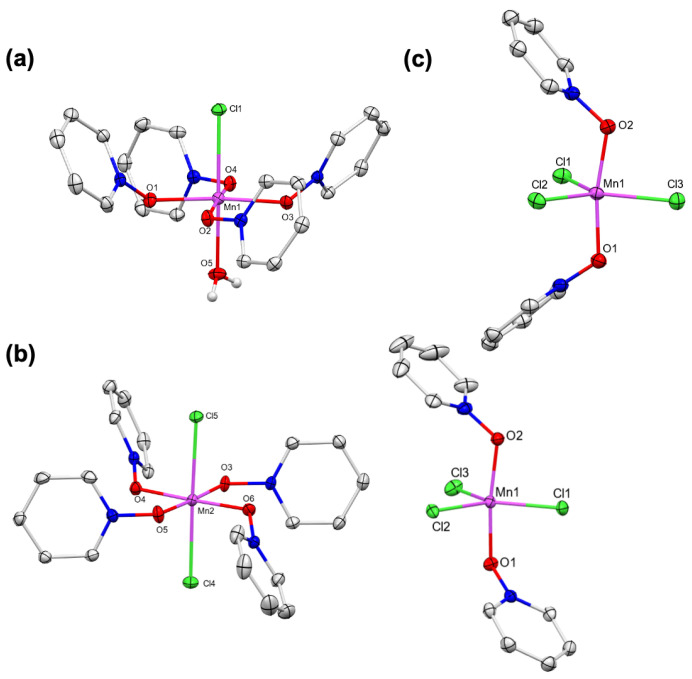
Molecular structures of Mn(III) centers in (**a**) **5**, (**b**) **6**·**7**, and (**c**) **7** (full crystal structures are presented in the SI). Selected bond lengths (Å) and angles (deg.) for **5**: Mn1–Cl1 = 2.5535(4); Mn1–O1 = 1.9389(12); Mn1–O2 = 1.9293(11); Mn1–O3 = 1.9301(12); Mn1–O4 = 1.9255(11); Mn1–O5 = 2.2340(13); O1–Mn1–Cl1 = 93.54(4); O1–Mn1–O4 = 92.03(5); O4–Mn1–Cl1 = 89.60(4). Selected bond lengths (Å) and angles (deg.) for **6**·**7**: Mn1–Cl1 = 2.2804(6); Mn1–Cl2 = 2.3687(6); Mn1–Cl3 = 2.2790(6); Mn1–O1 = 1.9218(15); Mn1–O2 = 1.9194(16); Mn2–Cl4 = 2.5172(6); Mn2–Cl5 = 2.5390(6); Mn2–O3 = 1.9397(15); Mn2–O4 = 1.9309(15); Mn2–O5 = 1.9493(15); Mn2–O6 = 1.9465(15); Cl1–Mn1–Cl2 = 116.23(2); Cl2–Mn1–Cl3 = 105.08(2); Cl1–Mn1–Cl3 = 138.69(3); O1–Mn1–Cl1 = 90.58(5); O2–Mn1–Cl1 = 84.49(5); O3–Mn2–Cl4 = 91.04(5); O3–Mn2–O4 = 89.02(6); O4–Mn2–Cl4 = 91.28(5). Selected bond lengths (Å) and angles (deg.) for **7**: Mn1–Cl1 = 2.3172(8); Mn1–Cl2 = 2.3091(8); Mn1–Cl3 = 2.2961(8), Mn1–O1 = 1.912(2); Mn1–O2 = 1.916(2); Cl1–Mn1–Cl2 = 109.02(3); O1–Mn1–O2 = 168.97(9).

The FTIR spectrum of **5** exhibits a shifted N–O stretch at 1199 cm^−1^ and O–H stretch at 3396 cm^−1^; the N–O stretch for free PyNO is 1238 cm^−1^. The Mn(III) center exhibits an octahedral geometry with trans chloride and aquo ligands. The complex exhibits Jahn teller distortion with elongation along the Mn–Cl axis (Figure 3a). Bond lengths and angles of the second manganese center are consistent with [Mn^II^Cl_4_]^2−^ [27]. The red–brown crystals of **5** dissolve in DCM or MeCN to produce deep green solutions which are stable in air. The UV-vis spectrum of **5** in MeCN exhibits three main bands centered at 332 nm (ε = 7200 M^−1^cm^−1^), 375 nm (ε = 6300 M^−1^cm^−1^), and 615 nm (ε = 820 M^−1^cm^−1^). 

When the reaction of **2** with PyNO was performed excluding air and moisture, we instead obtained the composite compound complex salt [Mn^III^(PyNO)_4_Cl_2_]_2_[Mn^II^Cl_4_]·[Mn^III^(PyNO)_2_Cl_3_] (**6**·**7**) (Scheme 3), which was characterized by XRD (Figure 3b). The molecular cation in **6**, [Mn^III^(PyNO)_4_Cl_2_]^+^, exhibits elongated trans-Mn–Cl bonds as a result of Jahn–Teller distortion and is charge-balanced with a [Mn^II^Cl_4_]^2–^. The neutral complex, [Mn^III^(PyNO)_2_Cl_3_] (**7**), has a distorted trigonal bipyramidal geometry (τ_5_ = 0.55), with the chloride ligands occupying the three equatorial positions and the PyNO ligands occupying the two axial positions (Figure 3c). The MeCN solution of **6**·**7** shows an intense green color with similar bands as **5** in the UV-vis spectrum at 340 nm (ε = 17,000 M^−1^cm^−1^), 375 nm (ε = 15,000 M^−1^cm^−1^), and 612 nm (1900 M^−1^cm^−1^).

The synthesis of **5** and **6**·**7** show that small changes in the reaction conditions lead to dramatically different results. In our hands, we found that the isolation of [Mn^III^(PyNO)_2_Cl_3_] (**7**) was only possible by treating **1** with two equivalents of PyNO in THF. Of the compounds described so far, **7** is the only thermally stable Mn(III) complex and thereby enabled us to determine the magnetic properties of the Mn(III) ion in isolation from any other magnetically active contaminant (The instability of **3**, the inclusion of Mn^II^ counterions in **5**, and the composite nature of **6**·**7** precluded similar characterization. Therefore, the magnetic properties of the other compounds were not pursued in this study. Generally, mononuclear Mn(III) centers studied by us have had S = 2 ground states. Some exceptions are strong-field six-coordinate cationic Mn(III) complexes that are S = 1 (see [7,11,13,17])). The Evans method indicates a high spin Mn(III) center (S = 2), consistent with its Mn(III) oxidation state assignment and similar to **1** and the other Mn(III) complexes we have prepared [18,22,23,24,34]. The FTIR spectrum of **7** exhibits a characteristic N–O stretch at 1188 cm^−1^ enabling a tentative assignment of 1201 cm^−1^ of the N–O stretch in **6** (Figure S14). The complex **7** dissolves in DCM and MeCN to produce intense green solutions with UV-vis bands centered at 340 nm (ε = 5100 M^−1^cm^−1^), 375 nm (ε = 4800 M^−1^cm^−1^), and 632 nm (ε = 1200 M^−1^cm^−1^). The Mn(III) center in **7** has a distorted trigonal bipyramidal geometry (τ_5_ = 0.70), with chloride and aquo ligands trans to each other similar to the structure found in **6**·**7** (Figure 3c). Under no set of conditions were we able to prepare the complex [Mn^III^Cl_3_(PyNO)_3_] [21].

Although the solid-state structures of **5**, **6**·**7**, and **7** are different, the UV-vis features in solution are strikingly similar. Likewise, the reduction potentials obtained from cyclic voltammetry (CV) and differential pulse voltammetry (DPV) are essentially the same (Figures S19–S21), each having a reversible reduction 0.47 ± 0.02 V vs. FeCp_2_ in MeCN (0.5 M [*n*Bu_4_N][PF_6_]). This implies that the differences are mostly due to conditions related to crystallization. We suspect that the solution state behavior is an equilibrated mixture of these species, although we do not know which is the dominant form in solution. This hypothesis is supported by their similar reactivity, which is described next.

### 2.4. Reactivity of Mn(III) Chloride Compounds

As noted above, we have shown that the C–H bonds can act as reductants toward Mn(III)X_3_ species [18,34]. Hence, we sought to explore this mode of reactivity of the newly synthesized Mn(III) chloride complexes with hexamethylbenzene (HMB) as the substrate. The mechanism for this reaction is consistent with C–H cleavage by an Mn^III^Cl species to form HCl and a benzylic radical [35,36,37,38,39], the latter of which rebounds with a second equivalent of Mn^III^Cl to form the C–Cl bond in the final product [40,41]; this mechanism is being studied in a separate study and is not discussed further here. Therefore, two equivalents of the Mn(III) reactant are needed for each C–H bond reacted; yields are reduced by 50% if only one equivalent of Mn(III) reactant is used. 

Complexes **3a**, **5**, and **7** were chosen for exploring the C–H chlorination reactivity for their combined ease of synthesis and handling and their well-defined Mn(III) stoichiometry. Each complex reacts with HMB to give the chlorinated product 1-cloromethyl-2,3,4,5,6-pentamethylbenzene (**8**). Out of all the complexes explored in this study, the pyridine *N*-oxide complexes produced the highest yield of **8**, reaching 88% conversion in 4 h for **7** and 86% in 6 h for **5** (Table 1). The similar reactivity of **5** and **7** supports the noted common solution–state speciation hypothesis. The reaction of HMB with **3a** produced **8** in only 40% yield; the Mn product is not **4** but some other Mn(II) byproduct. We suspect that the low yield is due to the insolubility of **3a** and competing side reactions, and **4** is unreactive toward chlorination reactivity, consistent with its Mn(II) oxidation state.

In comparing the reactivity between **7** and **1**, we noted that **1** is far more efficient at the chlorination of HMB, and **1** produces a near quantitative conversion to **8** at room temperature in 7 h [34]. This is consistent with the higher potential of **1**, which is 0.77 V vs. FeCp_2_ compared to 0.47 V for the PyNO complexes. Using the reduction potential of 0.47 V and a p*K*_a_ of 10.6 for HCl in MeCN [42] in the Bordwell equation furnishes an upper limit of C–H cleaving ability (i.e., BDFE_Mn(II)/X–H_ (Explanation for BDFE_Mn(II)/XH_. In a previous report [11], we used the reduction potential of [Mn^III^(NO_3_)_3_(OPPh_3_)_2_] and the p*K*_a_ of the conjugate acid of dissociated [NO_3_]^−^ in the Bordwell equation to arrive at a thermodynamic value and referred to it as an effective bond dissociation free energy (BDFE_eff_). The BDFE_eff_, described by Mayer [23], uses the reduction potential and p*K*_a_ of oxidant/base pairs that can combine in a single entity (e.g., through H-bonded adduct) to react in bimolecular C–H bond cleavage. The BDFE_eff_ can be used as an estimate for the upper-limit of C–H bond strength the oxidant/base pair can cleave. However, since the base (X) is coordinated to the Mn(III) center, it is more appropriate to use a {Mn^II^X–H} BDFE (BDFE_Mn(II)/XH_) like the {Mn^III^O–H} BDFE (BDFE_O–H_) reported in metal-oxo/metal-hydroxo conversions as described by Borovik and others [23]. Therefore, we use the same approach as Borovik except that the p*K*_a_ of the conjugate acid of the free base is used instead of the p*K*_a_ of [Mn^II^X_2_(HX)]/[Mn^II^X_3_]^−^ and refer to it as the BDFE_Mn(II)/XH_. Hence, the BDFE_Mn(II)/XH_ is an estimate of the upper limit of C–H bond strength that can be cleaved by a {Mn^III^X} reactant. We have performed a systemic analysis of this square scheme approach to estimate C–H cleavage capability in a previous report [17]) [43]) of 78 kcal/mol in MeCN. This is just below the 81 kcal/mol C–H bond strength in HMB [44] and thus consistent with the need to heat the reactions to achieve conversion. By contrast, **1** has a BDFE_Mn(II)/X–H_ of 85 kcal/mol and is thus capable of reacting with HMB at r.t. [34]. 

The reactivity of HMB with **2** was also explored. Although the yields were higher for **2** compared to the other Mn(III) reactants, the product mixture was complicated by several byproducts. Furthermore, the reaction with **2** proceeded quickly at r.t., consistent with the higher degree of reactivity expected of this solvated entity that is not stabilized by a pnictogen–oxide ligand.

### 2.5. Reaction of 1 with 2,2,6,6-Tetramethyl-1-piperidinyloxy (TEMPO)

**Scheme 5:**
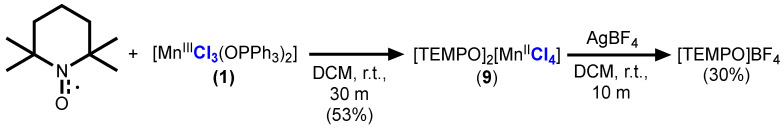
Reaction of **1** with TEMPO.

**Figure 4:**
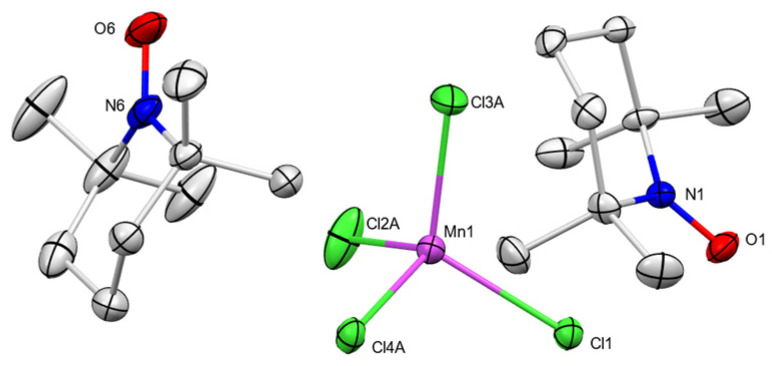
Molecular structure (ellipsoids 50%) of **9** determined with XRD (H atoms and one part of disorder omitted, only one of the three identical subunits in the unit cell shown for clarity) Selected bond lengths (Å) and angles (deg.) for **9**: Mn1–Cl1 = 2.3737(5); Mn1–Cl2A = 2.3549(18); Mn1–Cl3A = 2.3728(10); Mn1–Cl4A = 2.4025(15); N1–O1 = 1.1922(17); N6–O6 = 1.191(2); Cl1–Mn1–Cl2A = 108.62(6); Cl3A–Mn1–Cl4A = 103.78(6).

The reaction of **1** with TEMPO did not result in coordination of TEMPO to the manganese center but instead caused oxidation of TEMPO to form TEMPO^+^ ion (Scheme 5). This is not surprising given that the redox potential of **1** (0.77 V vs. FeCp_2_ in MeCN) is more positive than that of TEMPO (0.22 V vs. FeCp_2_) [45]. The orange product was characterized by XRD to reveal the *d*^8^ manganate salt of the TEMPO cation, [TEMPO]_2_[Mn^II^Cl_4_] (**9**) (Figure 4). No stretches associated with Ph_3_PO were observed in the FTIR spectrum of **9**. The compound has low solubility, but CHN and pXRD support the assignment of the bulk material as **9**. Treatment of this solid with AgBF_4_ allowed the isolation of a known salt [TEMPO]BF_4_ [46].

## 3. Conclusions

In this work, we set out to synthesize Mn(III) chloride complexes stabilized by ligands with *N*-oxide functional groups. The coordination of *N*-oxide ligands is rare, and we formed the first examples of Mn(III) stabilized by Me_3_NO ligands through the characterization of **3a** and **3b**. The species in **3a** was not characterized by diffraction techniques due to its instability. However, it decomposed into a unique molecular polymer salt, where one polymer was a cationic species with Me_3_NO coordination to Mn(II) centers in **4**. Hence, it is apparent that Me_3_NO can act as a ligand and not just as an O-atom transfer reagent as it is typically employed.

The synthesis and characterization of the PyNO complexes **5**, **6**·**7**, and **7** was also performed. Although the solid-state characterization for each revealed different molecular structures, their solution-state properties were essentially identical. As a result, their reactivity in solution with HMB was also nearly identical. They were, however, diminished in reactivity compared to **1** and this is rationalized by the lower reduction potentials for the PyNO complexes. In contrast, the highly reactive solvated entity in Christou’s solution (i.e., **2**) reacted with HMB rapidly at r.t., indicating that the coordination of PyNO remains in solution and has a stabilizing affect. Finally, when the potential of the ligand is low enough, electron transfer occurs as opposed to coordination. This was demonstrated with the stable radical TEMPO, which furnished outer sphere [TEMPO]^+^ ions rather than Mn^III^–TEMPO adducts.

## 4. Materials and Methods

**General Considerations:** All chemicals were purchased from chemical vendors and used as received unless otherwise noted. Anhydrous Me_3_NO was obtained by refluxing the dihydrate in toluene using a Dean–Stark apparatus on a Schlenk line overnight and subsequently isolating the material by removal of toluene. The solid thus obtained was washed with hexane, filtered, and dried under vacuum inside a glovebox. Material obtained this way contained no evidence of water by NMR or FTIR. Dry, oxygen-free solvents were obtained from a PPT solvent purification system, and were stored over 3 Å molecular sieves prior to use. Unless otherwise stated, synthetic manipulations of air sensitive compounds were performed in a nitrogen-filled VAC glovebox or on a Schlenk line. NMR experiments were carried out on Bruker Neo-400 MHz or Bruker Neo-500 MHz spectrometers. ATR-FTIR spectra were collected using a Bruker Alpha IR spectrometer with the “ATR Platinum” insert adapter (diamond crystal) stored inside a nitrogen-filled VAC Atmospheres glovebox. UV-vis experiments were performed using an 8154 Agilent Spectrophotometer equipped with an Unisoku cryostat. CHN combustion analyses were performed using a Thermo Scientific FlashEA1112 CHNS analyzer. Electrochemistry was performed on a SP-200 Bio-Logic potentiostat. The following compounds were prepared according to the literature: [Mn_12_O_l2_(OAc)_16_(H_2_O)_4_]·2HOAc·4H_2_O (**Mn_12_**) [47], [MnCl_3_(OPPh_3_)_2_] (**1**) [18]. 

**Crystallographic methods:** Low-temperature X-ray diffraction data for [Mn^III^Cl_2_(Me_3_NO)_3_]Cl·MeCN (**3b**) (CCDC No. 2382837), [Mn^III^Cl(H_2_O)(PyNO)_4_][Mn^II^Cl_4_] (**5**) (CCDC No. 2382830), [Mn^III^Cl_2_(PyNO)_4_]_2_[Mn^II^Cl_4_]·[Mn^III^Cl_3_(PyNO)_2_] (**6**·**7**) (CCDC No. 2382831), [Mn^III^Cl_3_(PyNO)_2_] (**7**) (CCDC No. 2382835), and (TEMPO)_2_[Mn^II^Cl_4_] (**9**) (CCDC No. 2382836) were collected on a Rigaku XtaLAB Synergy diffractometer coupled to a Rigaku HyPix detector with either Cu Kα radiation (λ = 1.54184 Å) or Ag Kα radiation (λ = 0.56087 Å, for **9**), from PhotonJet micro-focus X-ray sources at 100 K. The diffraction images were processed and scaled using the CrysAlisPro software v. 43.121 [48]. The structures were solved through intrinsic phasing using SHELXT [49] and refined against F2 on all data by full-matrix least squares with SHELXL [50], following established refinement strategies [51]. All non-hydrogen atoms were refined anisotropically. All hydrogen atoms bound to carbon were included in the model at geometrically calculated positions and refined using a riding model. The isotropic displacement parameters of all hydrogen atoms were fixed to 1.2 times the Ueq value of the atoms to which they are linked.

Electron diffraction measurements were performed on **4** (CCDC No. 2383143), using a Rigaku XtaLAB Synergy-ED equipped with a Rigaku HyPix-ED detector optimized for operation in the Micro-ED experimental setup1. Datasets were collected at 100 K with a wavelength of 0.0251 Å using the Rigaku program CrysAlisPro-ED for simultaneous sample measurement and data processing [25]. Samples were prepared by grinding the powder between two glass slides to reduce particle size and then sweeping a 3 mm Cu TEM grid with a lacey carbon film into the ground powder. The grid was then mounted on an Elsa 698 cryo-sample holder from Gatan and cooled to 100 K. Finally, the sample holder was inserted into the diffractometer for analysis.

The crystallite used for data collection for both samples was a thin particle with the other two dimensions ranging between 0.3 µm and 1 µm. Using Olex2 [52], crystal structures were readily solved with the SHELXT [49] structure solution program, using Intrinsic Phasing and refined kinematically with the SHELXL [50] refinement package, using least squares minimization. All atoms were refined in anisotropic approximation. The hydrogen atoms were placed at their idealized position and refined as riding atoms. Hydrogen bond distances from neutron diffraction data were used for final refinements in SHELXL (using the command ‘neutronHdist’), as hydrogen bond distances observed from electron diffraction are closer to those observed from neutron diffraction than from X-ray diffraction.

**Electrochemistry experiments:** The Mn^III^/Mn^II^ reduction potentials of Mn(III) complexes were determined using cyclic voltammetry and differential pulse voltammetry experiments. The electrochemical cell was equipped with a glassy carbon working electrode, Ag/Ag^+^ (4 mM AgNO_3_ in MeCN, 0.5 M [nBu_4_N][PF_6_]) reference electrode with a CoralPor^TM^ separator, and a platinum auxiliary electrode. Then, a 0.5 M solution of [nBu_4_N][PF_6_] in MeCN was used as the supporting electrolyte. Scans were performed with internal resistance compensation (85%). The analyte concentration was 4 mM for **5** and **7** and 1.33 mM for **6**. Scan direction was cathodic (scan rate: 200 mV/s). The reference electrode was externally referenced to ferrocene at the beginning and end of the experiment. For differential pulse voltammetry, the step height was set to 10 mV, the pulse height was 100 mV, the pulse width was 25 ms, and the step width was 100 ms. Scan direction was cathodic. 

**Synthesis:**

*Synthesis of [Mn^III^Cl_3_(ONMe_3_)_2_]_n_ (***3a***) and [MnCl_2_(Me_3_NO)_3_]Cl·MeCN (***3b***). Route A:* In a 50 mL round bottom flask equipped with a stir bar, **1** (800 mg, 1.14 mmol, 1 eq.) was stirred in 25 mL MeCN to give a navy-blue suspension. Me_3_NO (209 mg, 2.78, 2.5 eq.) was added as a solid under vigorous stirring to form a purple precipitate. The reaction mixture was allowed to stir at room temperature for 30 min. Upon completion of the reaction, the reaction mixture was filtered through a medium porosity glass fritted funnel (medium frit) to obtain a purple solid (**3a**) and a deep blue filtrate. The solid **3a** was washed with 3 × 2 mL MeCN, followed by 2 × 2 mL diethyl ether, and dried under vacuum (178.3 mg, 51%). Storage of the deep blue filtrate at −35 °C overnight produced 48 mg (10%) of deep purple crystals of **3b**. 

*Route B:* **Mn_12_** (100.0 mg, 0.048 mmol, 1 eq.) was dissolved in 10 mL MeCN in a 20 mL scintillation vial equipped with a stir bar and stirred to give an intense coffee brown mixture. Me_3_SiCl (0.220 mL, 1.75 mmol, 36 eq.) was added dropwise via syringe with vigorous stirring to give a deep purple solution containing **2**. Me_3_NO (87.5 mg, 1.16 mmol, 24 eq.) was added to the reaction mixture. The reaction mixture immediately turned deep green and formation of a purple precipitate was observed. After 30 min of stirring at room temperature, the reaction mixture was filtered through a medium frit and the solid was washed with MeCN (2 × 1 mL) and 1 mL diethyl ether, then dried under vacuum to yield the product **3a** as a purple solid (77.8 mg, 43%). Spectroscopic characterization matched the product obtained from route A.

ATR-FTIR (cm^−1^) of **3a**: 3050, 3033, 3019, 1483, 1460, 1390, 1265, 1234, 1118, 1052, 939, 921, 756, 579, and 479.

ATR-FTIR (cm^−1^) of **3b**: 3033, 3015, 2961, 2916, 2251, 1493, 1464, 1433, 1382, 1267, 1238, 1124, 968, 939, 931, 760, 581, 533, 484, and 472.

CHN-Analysis [calc. (found)] for **3a**, [Mn_3_Cl_9_(Me_3_NO)_5.7_]: %C22.09 (22.04), %H 5.56 (5.28), and %N 8.59 (8.49). The formula is presented as [MnCl_3_(Me_3_NO)_2_]_n_ throughout the manuscript for clarity.

*Synthesis of [Mn^II^(µ-Cl)_3_Mn^II^(µ-ONMe_3_)]_n_[Mn^II^(µ-Cl)_3_]_n_·(Me_3_NO·HCl)_3n_ (***4***).* Complex **3a** was added to a 20 mL scintillation vial and exposed to air overnight. The solid immediately turned brown within a minute of exposure to air to form **4** almost quantitatively. The same process occurred more slowly upon storage in the glovebox and could not be prevented, even at −35 °C.

ATR-FTIR (cm^−1^): 3033, 2785, 2704, 2663, 2626, 1522, 1485, 1468, 1452, 1432, 1394, 1256, 1124, 950, 750, 486, 453, and 430.

CHN-Analysis [calc. (found)] for [MnCl_2_(ONMe_3_)]·(Me_3_NO·HCl): %C 23.06 (23.58), %H 6.13 (6.38), and %N 8.96 (8.83).

*Synthesis of [MnCl(H_2_O)(PyNO)_4_][MnCl_4_] (***5***). Route A:* **Mn_12_** (100 mg, 0.048 mmol, 1 eq.) was dissolved in 20 mL MeCN in a Schlenk flask under Ar atmosphere and stirred to give an intense coffee-brown solution. Me_3_SiCl (0.220 mL, 1.75 mmol, 36 eq.) was added dropwise via syringe with vigorous stirring to give a deep purple mixture containing **2**. PyNO (110 mg, 1.16 mmol, 24 eq.) was added in one portion to the reaction mixture. The reaction mixture immediately turned deep emerald green. After 30 min of stirring at room temperature, the reaction mixture was reduced to 5 mL in vacuo. The solution was filtered through a medium frit, transferred (open to air) into a 25 mL Erlenmeyer flask, and left undisturbed for slow evaporation at room temperature for 5 days, from which 109 mg (56% yield) of deep red–brown crystals of **5** was isolated.

*Route B:* This procedure was performed open to air. In a 20 mL scintillation vial, PyNO (212 mg, 2.23 mmol, 2 eq.) was dissolved in 40 mL 200 proof ethanol at room temperature. Under vigorous stirring, **1** (800 mg, 1.11 mmol, 1 eq.) was added as a solid to cause an immediate formation of a green reaction mixture and formation of a brown precipitate. The reaction mixture was allowed to stir for 30 min. The mixture was then filtered through a medium frit to isolate a brown-black residue. The residue was extracted with 3 × 10 mL EtOH. The EtOH washings were combined and poured into 70 mL pet. ether to precipitate **5** as a red–brown solid. The solid was washed with 10 mL pet. ether and dried open to air (185.6 mg, 51%). Spectroscopic characterization matches the product obtained via route A.

ATR-FTIR (cm^−1^): 3112, 3051, 1464, 1200, 1171, 1095, 1070, 1023, 830, 770, and 667.

^1^H-NMR (CD_2_Cl_2_): 1.7, 10.4, 14.7, and 20.0.

CHN-Analysis [calc. (found)] for [MnCl(H_2_O)(PyNO)_4_][MnCl_4_]·H_2_O: %C 34.14 (33.82), %H 3.44 (3.38), and %N 7.96 (7.81).

UV-vis λ_max_ [MeCN, nm (ε, M^−1^cm^−1^)]: 332 (7200), 375 (6300), and 615 (820).

*Synthesis of [MnCl_2_(PyNO)_4_]_2_[MnCl_4_]·[Mn(PyNO)_2_Cl_3_] (***6·7***).***Mn_12_** (100 mg, 0.048 mmol, 1 eq.) was dissolved in 10 mL MeCN in a 20 mL scintillation vial equipped with a stir bar and stirred to give an intense coffee-brown mixture. Me_3_SiCl (0.220 mL, 1.75 mmol, 36 eq.) was added dropwise via syringe with vigorous stirring to give a deep purple solution containing **2**. PyNO (110 mg, 1.16 mmol, 24 equiv.) was added in one portion to the reaction mixture. The reaction mixture immediately turned deep emerald green. After 30 min of stirring at room temperature, the reaction mixture reduced to 5 mL under vacuum. The solution was filtered through a medium frit and stored at −35 °C for slow evaporation, from which 84.0 mg (36% yield) of deep green crystals of **6**·**7** was isolated.

ATR-FTIR (cm^−1^): 3112, 3051, 1469, 1198, 1174, 1095, 1071, 1022, 834, 824, 774, and 667.

^1^H-NMR (CD_2_Cl_2_): 102, 13.2, and 18.0.

CHN-Analysis [calc. (found)] for [MnCl_2_(PyNO)_4_]_2_[MnCl_4_]·[Mn(PyNO)_2_Cl_3_]: %C 38.48 (38.18), %H 3.23 (3.31), and %N 8.97 (8.98).

UV-vis λ_max_ [MeCN, nm (ε, M^−1^cm^−1^)]: 340 (17,000), 375 (15,000), and 612 (1900).

*Synthesis of [MnCl_3_(PyNO)_2_] (***7***).* In a 20 mL scintillation vial equipped with a stir bar, **1** (200 mg, 0.279 mmol, 1 eq.) was stirred in 12 mL THF to give a purple suspension. PyNO (53 mg, 0.56, 2 eq.) was added as a solid under vigorous stirring to cause a color change to green. The reaction mixture was stirred at room temperature overnight (~16 h). Upon completion of the reaction, the reaction mixture was filtered through a medium frit and the product was obtained as a green solid that was washed with 2 × 1 mL THF, followed by 2 × 1 mL pentane, and dried under vacuum (94 mg, 95%). Crystals of **7** were obtained from slow diffusion of a saturated DCM solution of **7** with pet. ether at −35 °C.

ATR-FTIR (cm^−1^): 3116, 3077, 3050, 1606, 1464, 1244, 1188, 1166, 1096, 1073, 1042, 1026, and 933.

^1^H-NMR (CD_2_Cl_2_): 11.8, 17.9, 25.4.

Evans method (CD_3_CN, 500 MHz, 298 K) μ_eff_ = 4.96 μB.

CHN [calc. (found)] for [MnCl_3_(PyNO)_2_]·CH_2_Cl_2_: %C 30.27 (30.70), %H 2.77 (2.62), and %N 6.42 (6.82).

UV-vis λ_max_ [DCM, nm (ε, M^−1^cm^−1^)]: 340 (5100), 375 (4800), and 632 (1200).

*General procedure for chlorination of hexamethylbenzene using Mn–Cl complexes:* This procedure is modified after a literature report [34]. The Mn complex (0.0813 mmol, 2.2 eq of Mn(III)) was weighed out into a 25 mL bomb flask with a stir bar and 4 mL MeCN was added. A stock solution of HMB was prepared in DCM (74 mM). The HMB solution (0.5 mL or 6 mg, 0.0369 mmol, 1.0 eq.) was added to the reaction mixture and sealed and left to stir at 60 °C until the deep coloration of the solution disappeared. Upon completion of the reaction, the reaction mixture was reduced to ≈0.2 mL under vacuum and loaded onto a plug of silica (pipet, 1.5 inches) to remove metal containing byproducts. The plug was eluted with 15 mL DCM. To the combined DCM washings, an internal standard (10 mg of 2-nitro benzaldehyde) was added and then the solvent was removed under vacuum. The solid residue obtained was dissolved in CDCl_3_ to prepare an NMR sample. Average yields were calculated from duplicated trials.

*Reaction of ***2*** with hexamethylbenzene:* In a 20 mL scintillation vial equipped with a stir bar, **Mn_12_** (31.7 mg, 0.0154 mmol, 1 eq.) was stirred in 5 mL MeCN to give a coffee-brown mixture. Me_3_SiCl (0.070 mL, 0.554 mmol, 36 eq.) was added dropwise via a syringe with vigorous stirring to give a deep purple solution containing **2** and left to stir for 5 min at room temperature. HMB (15.0 mg, 0.0924 mmol, 6.0 eq.) was added as a solid to the reaction mixture. The reaction vessel was sealed and left to stir for one hour until the deep coloration of the solution disappeared. Upon completion of the reaction, the reaction mixture was reduced to ≈0.2 mL under vacuum and loaded onto a plug of silica (pipet, 1.5 inches) to remove metal-containing byproducts. The plug was eluted with 20 mL DCM. To the combined DCM washings, internal standard (10 mg, 2-nitro benzaldehyde) was added and then the solvent was removed under vacuum. The solid residue obtained was dissolved in CDCl_3_ to prepare NMR sample. 

*Synthesis of (TEMPO)_2_[MnCl_4_] (***9***):* In a 20 mL scintillation vial, **1** (300 mg, 0.418 mmol, 1 eq.) was stirred in 2 mL DCM to give a deep blue solution. A solution of TEMPO (65.3 mg, 0.418 mmol, 1 eq.) in DCM was added to the reaction mixture under vigorous stirring. The reaction mixture immediately turned red and gradually formed an orange precipitate. The reaction mixture was left to stir for 30 min at room temperature, then filtered through a medium frit to isolate **9** as an orange precipitate (56.6 mg, 53%). The precipitate was washed with 1 mL DCM and 1 mL pentane and dried under vacuum. The deep orange filtrate was reduced to ≈2 mL under vacuum and stored for slow diffusion with pentane at −35 °C to obtain a few orange crystals of **9.** Then, **9** was recrystallized from a solution of **9** in MeCN by slow diffusion of diethyl ether at −35 °C to obtain crystals suitable for XRD.

ATR-FTIR (cm^−1^) of **9**: 2991, 2945, 2877, 1608, 1460, 1394, 1380, 1332, 1293, 1239, 1215, 1211, 1147, 1116, 1098, 1067, 980, 941, 881, 863, 857, 764, 725, and 702.

ATR-FTIR (cm^−1^) of [TEMPO]BF_4_: 3002, 2965, 2939, 1627, 1473, 1460, 1398, 1382, 1289, 1240, 1213, 1098, 1044, 974, 945, 900, 877, 856, 762, and 704.

CHN [calc. (found)] for {(TEMPO)_2_[MnCl_4_]}_n_·0.1CH_2_Cl_2_: %C 41.99 (41.74), %H 7.05 (6.86), and %N 5.41 (5.83).

NMR (CD_3_CN) of [TEMPO]BF_4_: ^1^H (400 MHz) 2.41, 2.14, 1.65 ppm; ^19^F-{^1^H} (376 MHz) −151.85 ppm.

*Reaction of ***9 ***with AgBF_4_.* A stirring suspension of **9** in DCM was treated with AgBF_4_ (80 mg, 0.411 mmol, 1 eq.), which caused the immediate formation of a white solid (AgCl and MnCl_2_) and a yellow solution. The reaction mixture was filtered and the yellow filtrate was collected. Yellow crystals of [TEMPO]BF_4_ were obtained after removing volatiles from the filtrate under vacuum (30.0 mg, 30%). The spectroscopic data agree with the literature [46].

## Figures and Tables

**Scheme 4:**
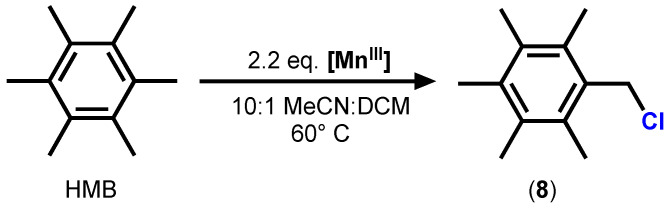
Conditions for C–H chlorination reactivity of HMB with Mn(III) chloride compounds.

**Table 1:** C–H chlorination reactivity of HMB with Mn(III) chloride compounds.

Complex	Time	% Yield ^(a)^
[Mn^III^Cl_3_(Me_3_NO)_2_]_n_ (**3a**)	5.0 h	45
[Mn^III^Cl(H_2_O)(PyNO)_4_][Mn^II^Cl_4_] (**5**)	6.5 h	86
[Mn^III^Cl_3_(PyNO)_2_] (**7**)	4.0 h	88
[Mn^III^Cl_3_(MeCN)_x_] (**2**)	1.0 h at r.t. ^(b)^	78
[Mn^II^(µ-Cl)_3_Mn^II^(µ-ONMe_3_)]_n_[Mn^II^(µ-Cl)_3_]_n_·(Me_3_NO·HCl)_3n_ (**4**)	5.0 h	0

See Scheme 4 for conditions. (a) Yields determined by ^1^H-NMR, average yields from duplicate runs reported; (b) reaction performed in MeCN without DCM added.

## Data Availability

The raw data supporting the conclusions of this article are available from the authors on request.

## Supplementary Materials

The following supporting information can be downloaded at: https://www.mdpi.com/article/10.3390/molecules29194670/s1, Figure S1: ATR-FTIR spectra of **3a** and **3b**; Figure S2: ATR-FTIR spectrum of **4**; Figure S3: PXRD spectra of **4** and authentic Me_3_NO·HCl; Figure S4: ^1^H-NMR spectrum of **5** in CD_2_Cl_2_; Figure S5: ATR-FTIR spectrum of **5**; Figure S6: UV-vis spectrum of **5** in MeCN at various concentrations with corresponding Beer’s law plot for extinction coefficient; Figure S7: molecular structure of **5** determined with XRD; Figure S8: ^1^H-NMR spectrum of **6**·**7** in CD_2_Cl_2_; Figure S9: ATR-FTIR spectrum of **6**·**7**; Figure S10: UV-vis spectrum of **6**·**7** in MeCN at various concentration with corresponding Beer’s law plot for extinction coefficient; Figure S11: molecular structure of **6**·**7** determined with XRD; Figure S12: ^1^H-NMR spectrum of **7** in CD_2_Cl_2_; Figure S13: ATR-FTIR spectrum of **7**; Figure S14: UV-vis spectrum of **7** in DCM at various concentration with corresponding Beer’s law plot for extinction coefficient; Figure S15: ATR-FTIR spectra of **9**, TEMPO, and (TEMPO)BF_4_; Figure S16: ATR-FTIR spectra of **6**·**7** and **7**; Figure S17: PXRD spectra of **9**; Figure S18: ^1^H-NMR spectrum of [TEMPO]BF_4_ in CD_3_CN; Figure S19: ^19^F-NMR spectrum of [TEMPO]BF_4_ in CD_3_CN; Figure S20: overlaid differential pulse voltammograms of **5**, **6**·**7**, and **7**; Figure S21: the cyclic voltammogram of **5** and **7** overlaid with the Mn^III^/Mn^II^ reversible event; Figure S22: the cyclic voltammogram of **6**·**7** overlaid with the Mn^III^/Mn^II^ reversible event; Figure S23: cyclic voltammograms of **5** at varying scan rates and peak current vs. (scan rate)^1/2^ with linear fit; Figure S24: cyclic voltammograms of **6**·**7** at varying scan rates and peak current vs. (scan rate)^1/2^ with linear fit; Figure S25: cyclic voltammograms of **7** at varying scan rates and peak current vs. (scan rate)^1/2^ with linear fit.
