# Supplementary material for: N-Oxide Coordination to Mn(III) Chloride
Source: Molecules. 2024 Oct 1;29(19):4670. doi: 10.3390/molecules29194670 (PMC11477729; doi:10.3390/molecules29194670)
Supplement: Supplementary file 1 [file molecules-29-04670-s001.zip › molecules-3224028-supplementary.pdf]

N-Oxide coordination to Mn(III) chloride

Ananya Saju[1], Dr. Matthew R. Crawley[1], Dr. Samantha N. MacMillan, Pierre[2], Le Magueres[3], Mark Del Campo[3] and § David C. Lacy[1]*

[1]*Department of Chemistry, University at Buffalo, State University of New York, Buffalo, New York 14260, United States.*
[2]*Department of Chemistry and Chemical Biology, Cornell University, Ithaca, New York 14853, United States.*
[3]*Rigaku Americas, The Woodlands, TX, USA*

Index

Contents **Page**

*Characterization of $[Mn^{III}Cl_3(ONMe_3)_2]_n$ (**3a**) and $[Mn^{III}Cl_2(ONMe_3)_3]Cl•MeCN$ (**3b**):* 2
*Characterization of $[Mn^{II}(\mu\text{-}Cl)_3Mn^{II}(\mu\text{-}ONMe_3)]_n[Mn^{II}(\mu\text{-}Cl)_3]_n•(Me_3NO•HCl)_{3n}$ (**4**)*........ 2
*Characterization of $[MnCl(H_2O)(OPy)_4][MnCl_4]$ (**5**):* 3
*Characterization of $[MnCl_2(OPy)_4]_2[MnCl_4]•[Mn(OPy)_3Cl_2]$ (**6•7**):* 4
*Characterization of $MnCl_3(PyNO)_2$ (**7**)* 6
*Characterization of $(TEMPO)_2[MnCl_4]$ (**9**) and $[TEMPO]BF_4$:* 8
Electrochemistry experiments 9

*Characterization of $[Mn^{III}Cl_3(ONMe_3)_2]_n$ (**3a**) and $[Mn^{III}Cl_2(ONMe_3)_3]Cl\bullet MeCN$ (**3b**):*

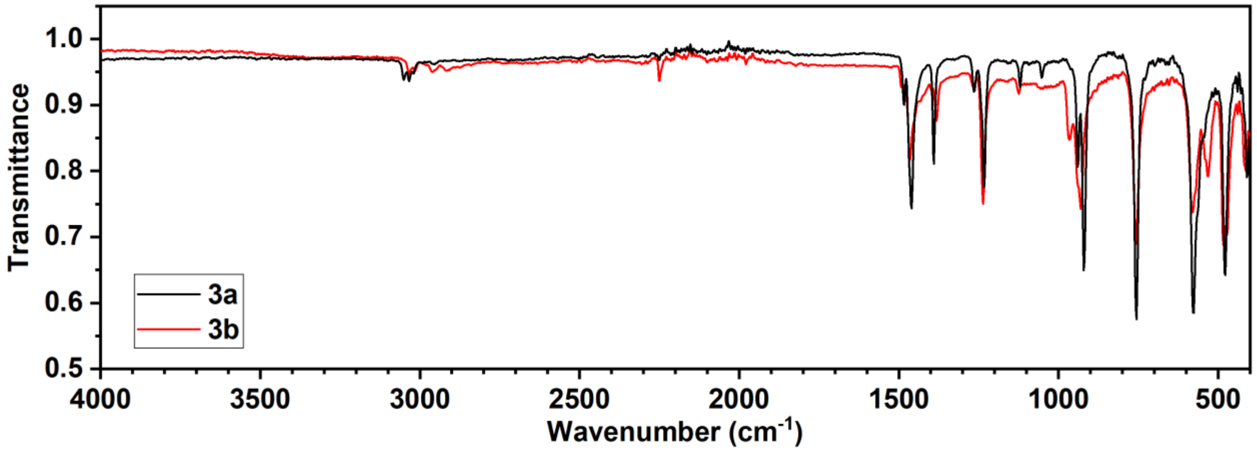

Figure S1: ATR-FTIR spectra of **3a** and **3b**.

*Characterization of $[Mn^{II}(\mu\text{-}Cl)_3Mn^{II}(\mu\text{-}ONMe_3)]_n[Mn^{II}(\mu\text{-}Cl)_3]_n\bullet(Me_3NO\bullet HCl)_{3n}$ (**4**)*

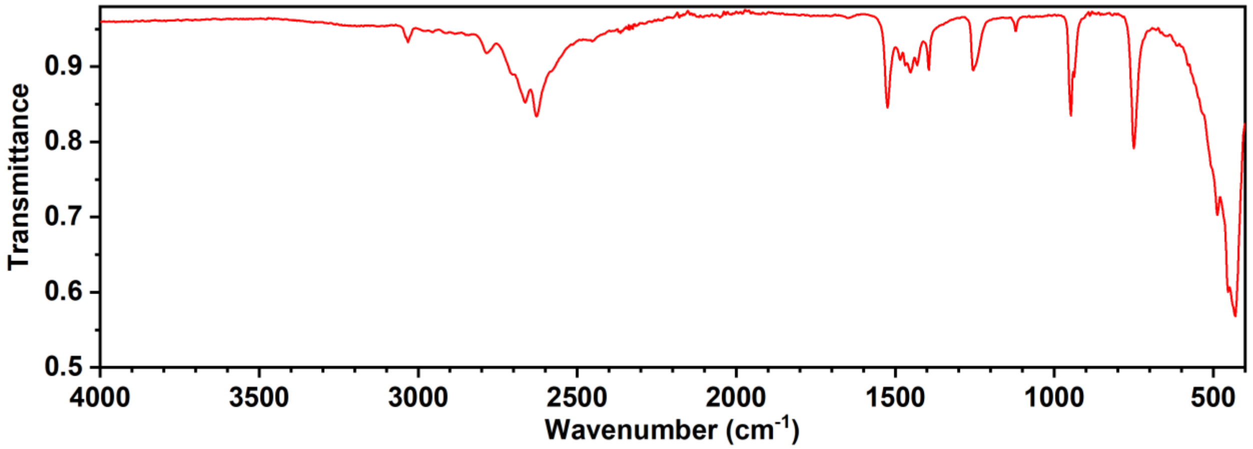

Figure S2: ATR-FTIR spectrum of **4**.

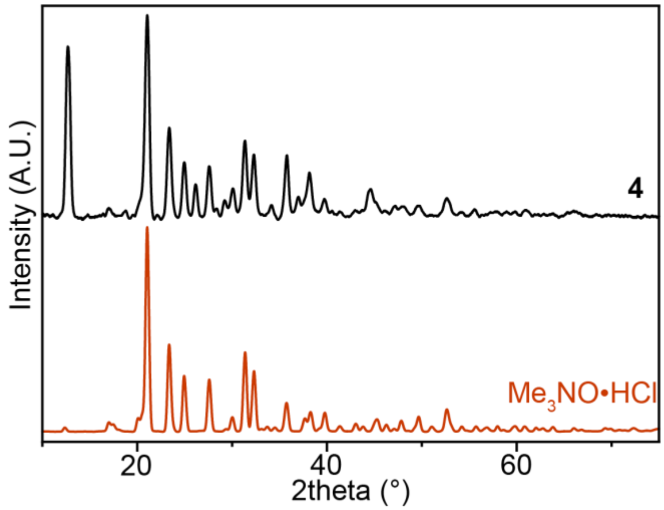

Figure S3: PXRD spectra of **4** and authentic $Me_3NO\bullet HCl$.

*Characterization of [$MnCl(H_2O)(OPy)_4$][$MnCl_4$] (**5**):*

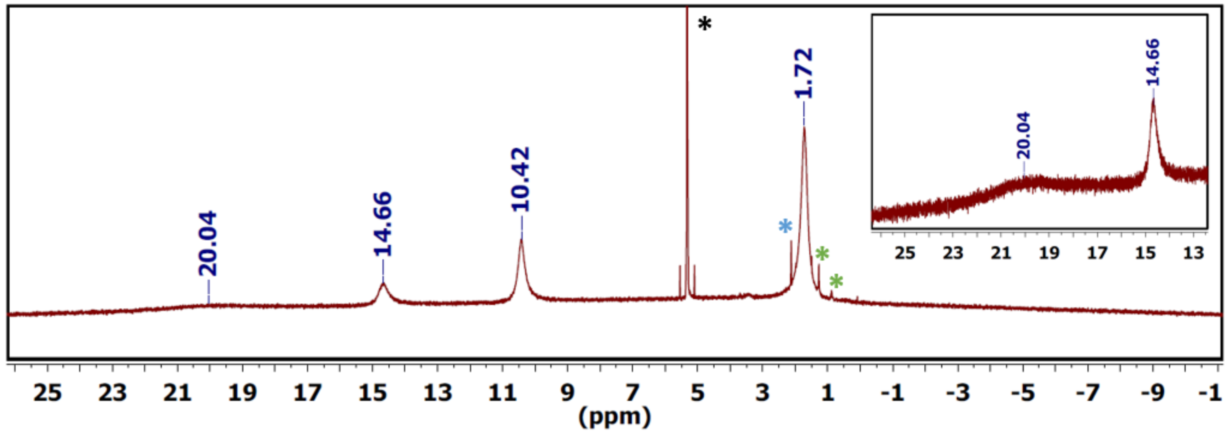

Figure S4: ^{1}H-NMR spectrum of **5** in CD_2Cl_2 (*).(*)indicates hexanes and (*) indicates acetone. Insert shows magnified spectra in 12-26 ppm region.

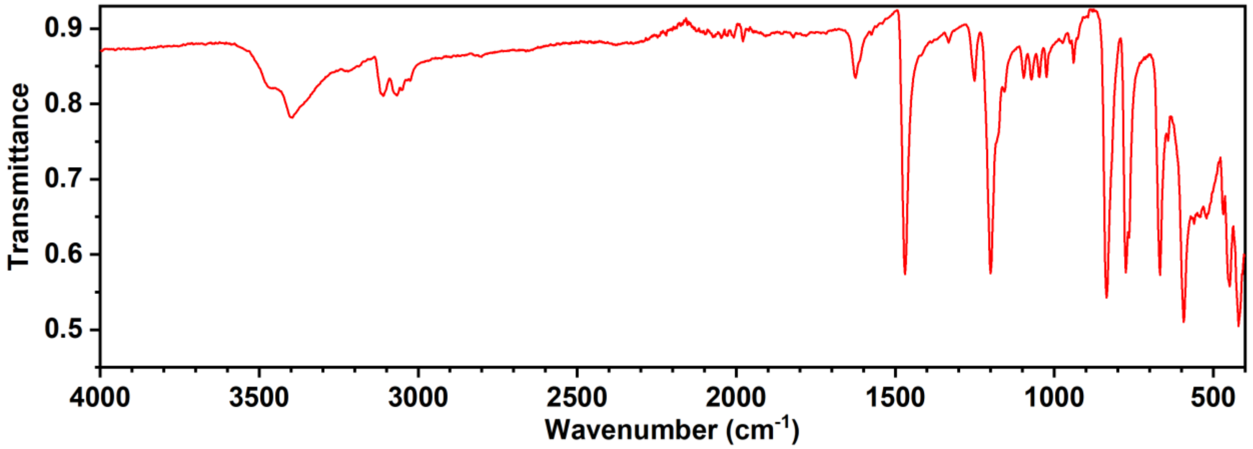

Figure S5: ATR-FTIR spectrum of **5**.

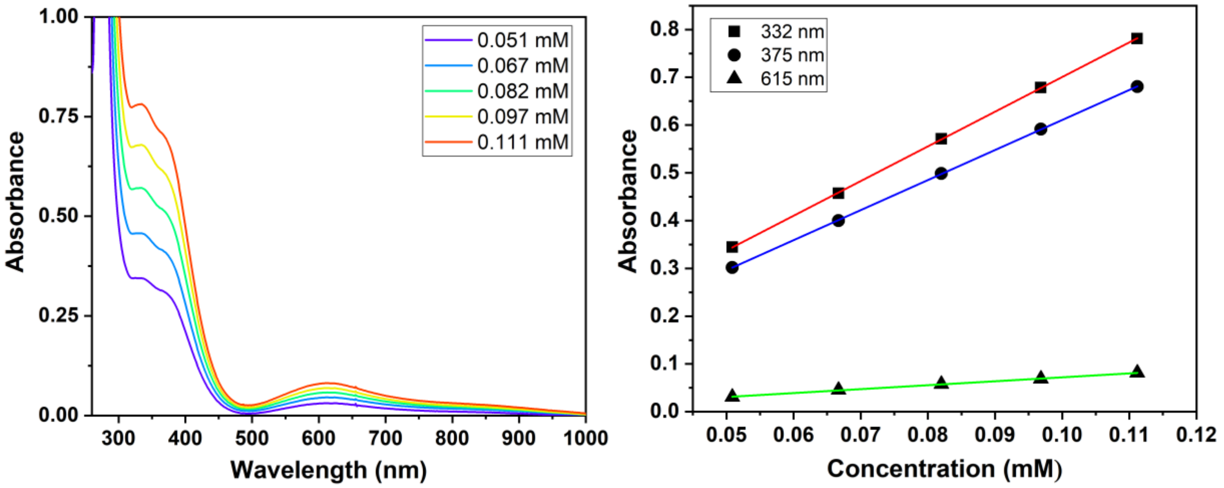

Figure S6: UV-vis spectrum of **5** in MeCN at various concentration (left) with corresponding Beer's law plot for extinction coefficient (right).

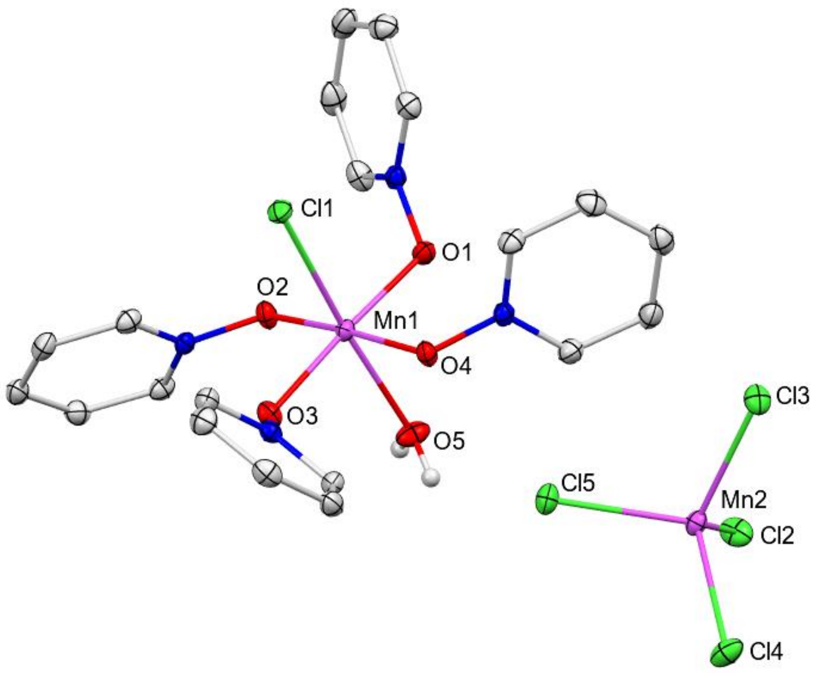

Figure S7: Molecular structure (ellipsoids 50%) of **5** determined with XRD (H atoms except two connected to O5 and one H_2O molecule omitted for clarity) Selected bond lengths (Å) and angles (deg.) for **5**: Mn1–Cl1 = 2.5535(4); Mn1–O1 = 1.9389(12); Mn1–O2 = 1.9293(11); Mn1–O3 = 1.9301(12); Mn1–O4 = 1.9255(11); Mn1–O5 = 2.2340(13); Mn2–Cl2 = 2.3526(5); Mn2–Cl3 = 2.3818(5); Mn2–Cl4 = 2.3454(5); Mn2–Cl5 = 2.3706(4); O1–Mn1–Cl1 = 93.54(4); O1–Mn1–O4 = 92.03(5); O4–Mn1–Cl1 = 89.60(4); Cl2–Mn2–Cl3 = 111.476(17); Cl2–Mn2–Cl4 = 114.175(17).

*Characterization of $[MnCl_2(OPy)_4]_2[MnCl_4]$•$[Mn(OPy)_3Cl_2]$ (**6•7**):*

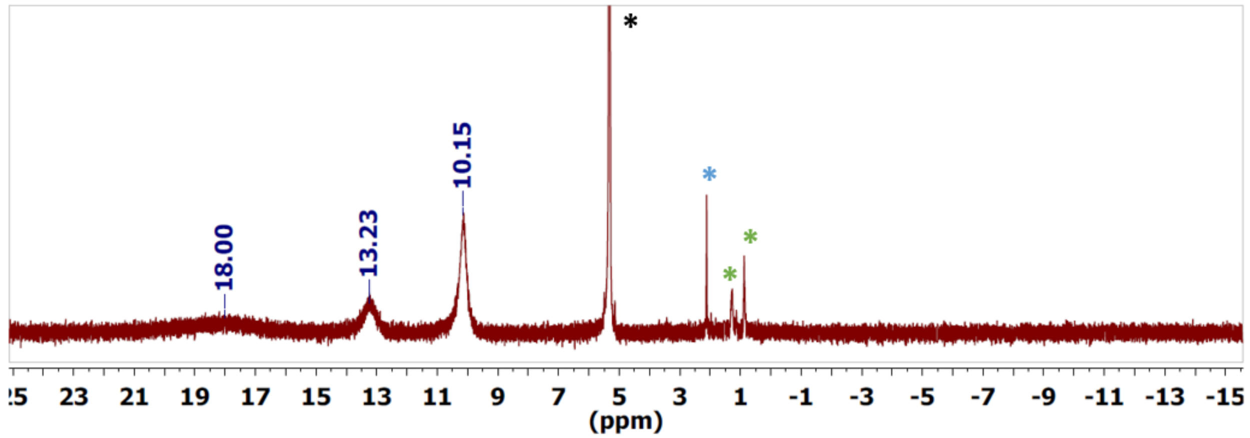

Figure S8: ^1H-NMR spectrum of **6•7** in CD_2Cl_2 (*).(*)indicates hexanes and (*) indicates acetone.

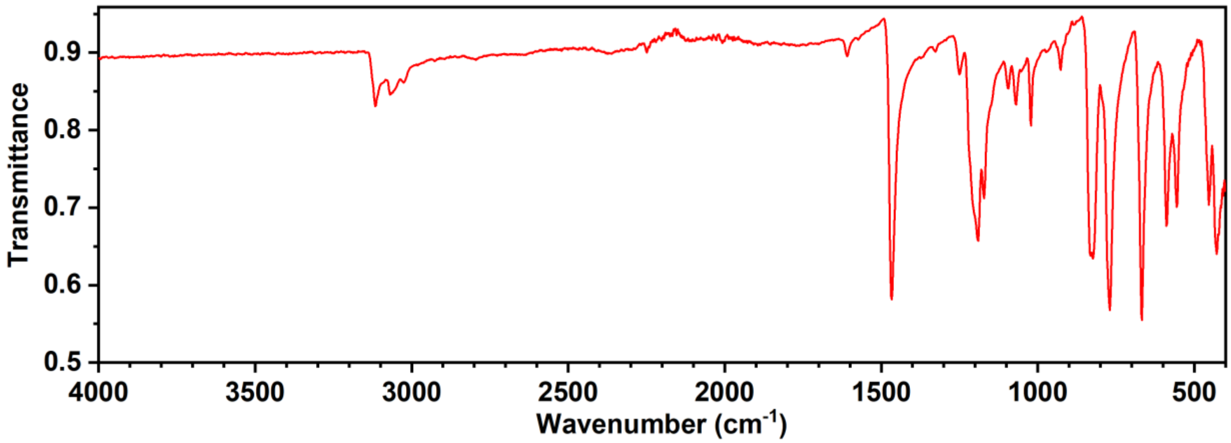

Figure S9: ATR-FTIR spectrum of **6•7**.

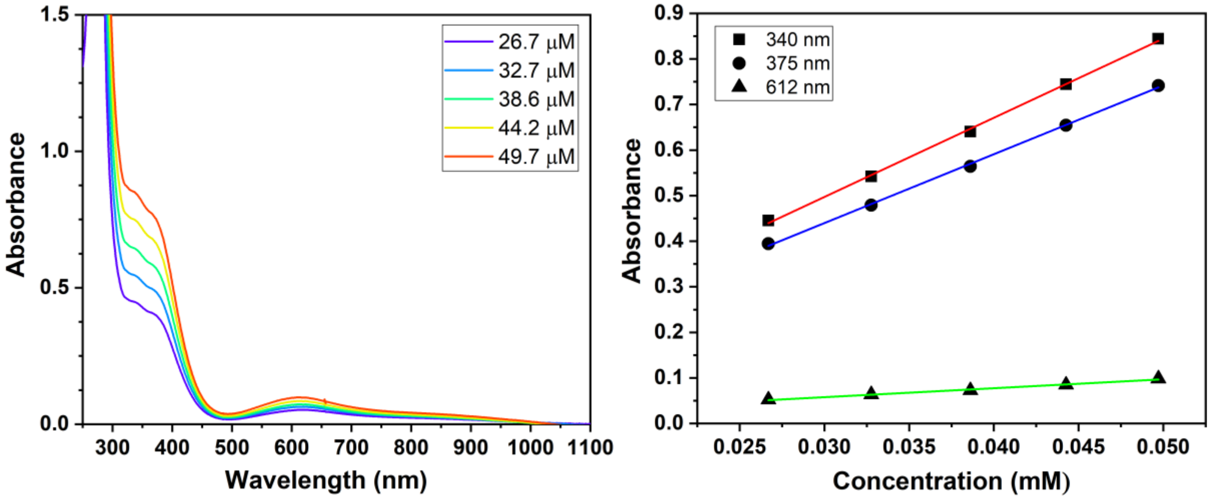

Figure S10: UV-vis spectrum of **6•7** in MeCN at various concentration (left) with corresponding Beer's law plot for extinction coefficient (right).

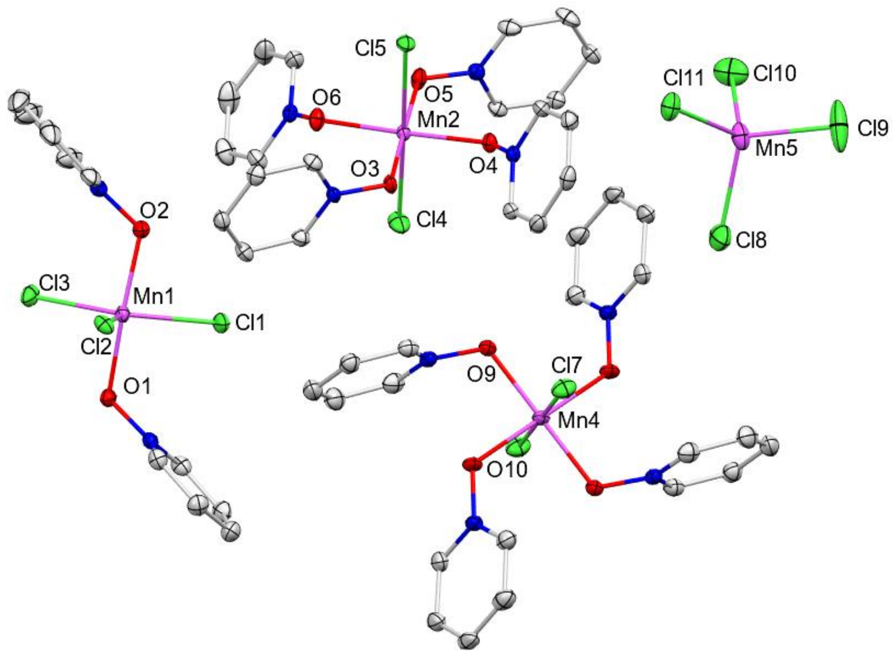

Figure S11: Molecular structure (ellipsoids 50%) of **6•7** determined with XRD (H atoms and one DCM molecule omitted for clarity) Selected bond lengths (Å) and angles (deg.) for **6•7**: Mn1–Cl1 = 2.2804(6); Mn1–Cl2 = 2.3687(6); Mn1–Cl3 = 2.2790(6); Mn1–O1 = 1.9218(15); Mn1–O2 = 1.9194(16); Mn2–Cl4 = 2.5172(6); Mn2–Cl5 = 2.5390(6); Mn2–O3 = 1.9397(15); Mn2–O4 = 1.9309(15); Mn2–O5 = 1.9493(15); Mn2–O6 = 1.9465(15); Mn5–Cl8 = 2.3542(8); Mn5–Cl9 = 2.3512(7); Cl1–Mn1–Cl2 = 116.23(2); Cl2–Mn1–Cl3 = 105.08(2); Cl1–Mn1–Cl3 = 138.69(3); O1–Mn1–Cl1 = 90.58(5); O2–Mn1–Cl1 = 84.49(5); O3–Mn2–Cl4 = 91.04(5); O3–Mn2–O4 = 89.02(6); O4–Mn2–Cl4 = 91.28(5); O9–Mn4–Cl7 = 90.11(5); O9–Mn4–O10 = 91.56(6); O10–Mn4–Cl7 = 90.69(5); Cl8–Mn5–Cl10 = 110.59(3); Cl8–Mn5–Cl11 = 108.36(3).

Characterization of $MnCl_3(PyNO)_2$ (7)

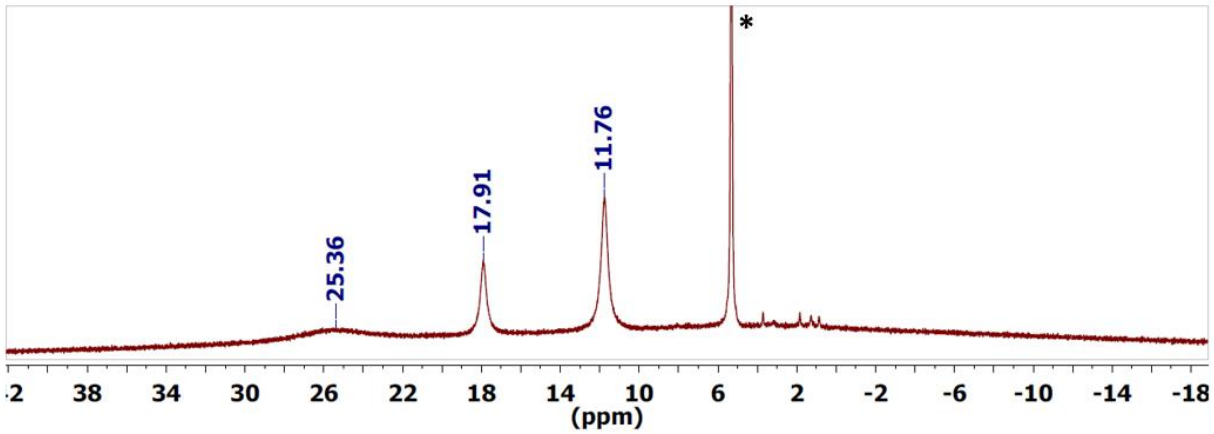

Figure S12: ^{1}H-NMR spectrum of **7** in CD_2Cl_2 (*).

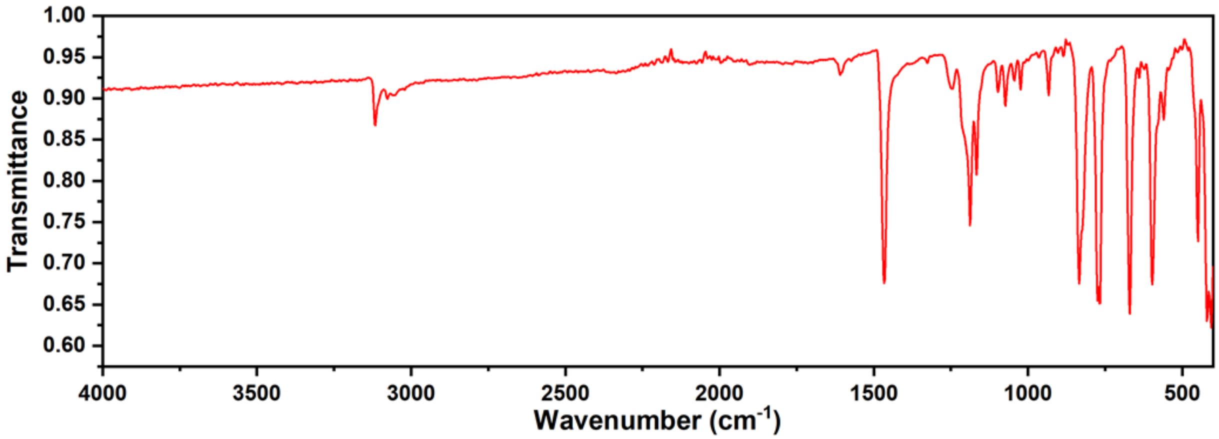

Figure S13: ATR-FTIR spectrum of **7**.

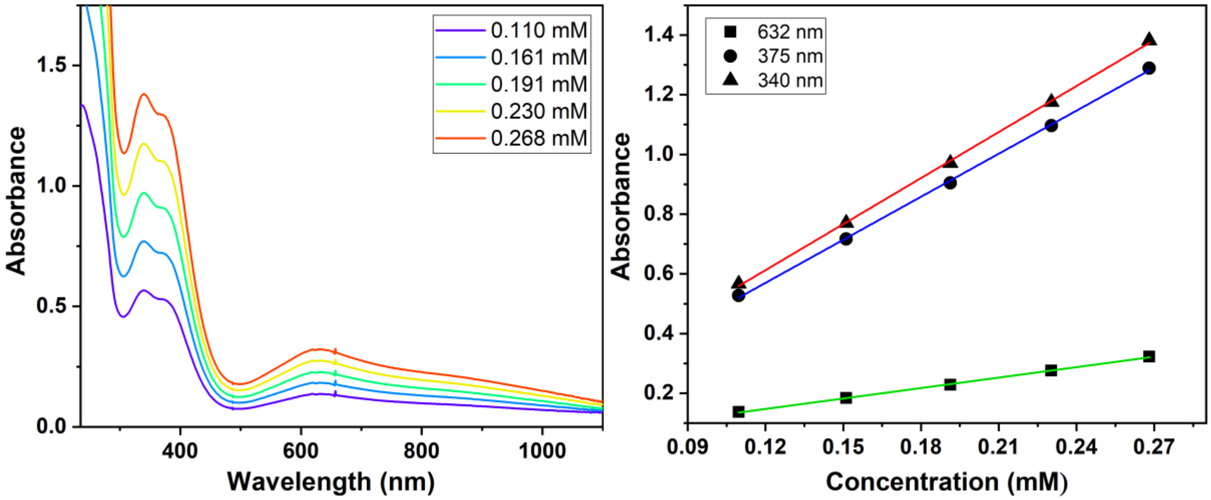

Figure S14: UV-vis spectrum of **7** in DCM at various concentration (left) with corresponding Beer's law plot for extinction coefficient (right).

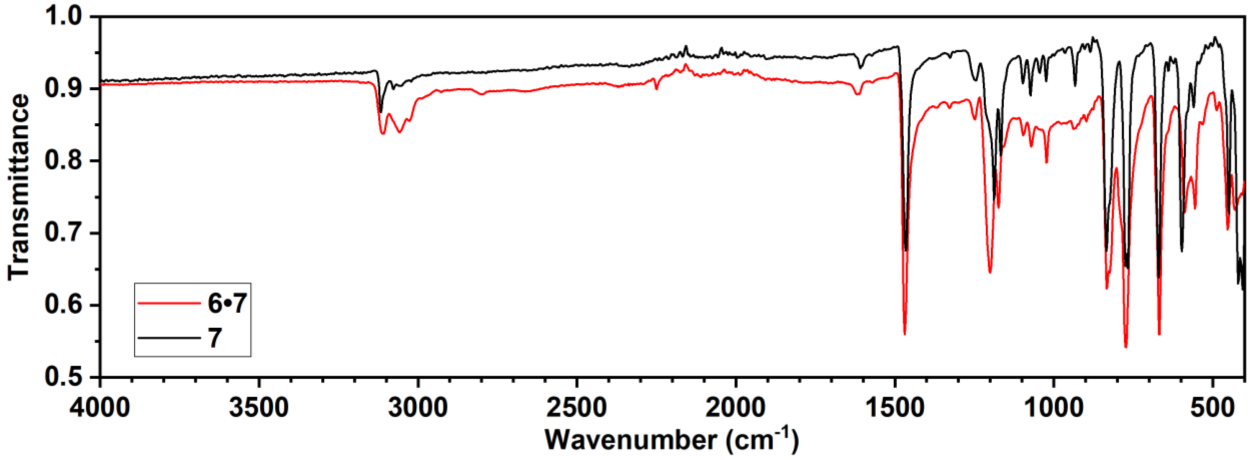

Figure S15: ATR-FTIR spectra of **6•7** and **7**.

*Characterization of $(TEMPO)_2[MnCl_4]$ (**9**) and $[TEMPO]BF_4$:*

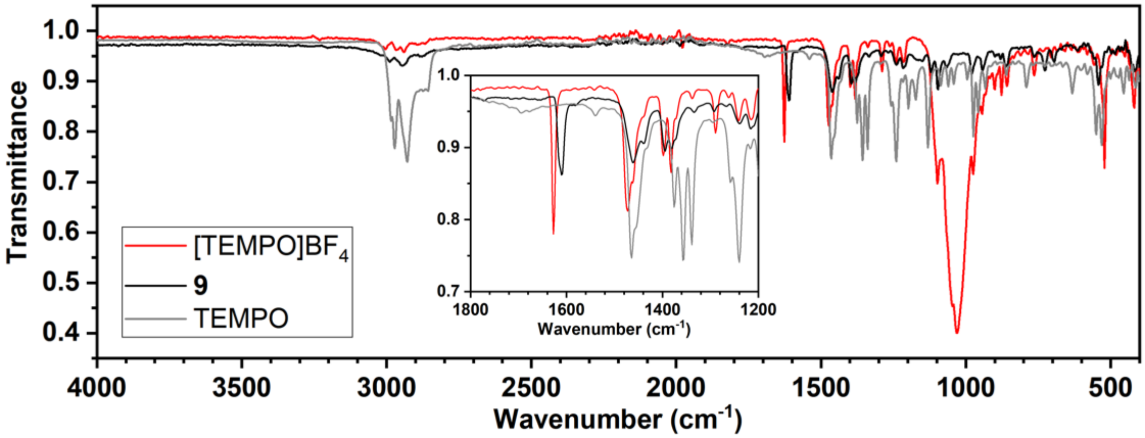

Figure S16: ATR-FTIR spectra of **9**, TEMPO, and $[TEMPO]BF_4$.

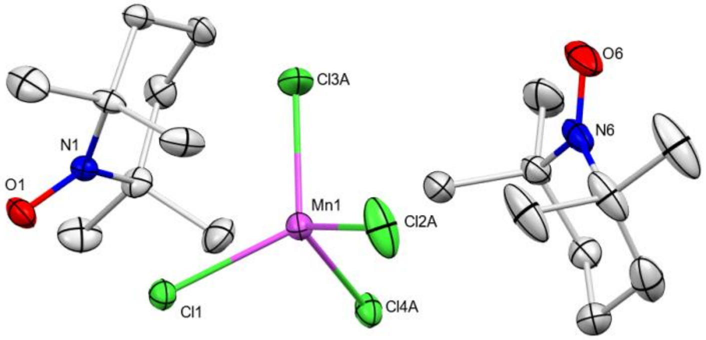

Figure S17: Molecular structure (ellipsoids 50%) of **9** determined with XRD (H atoms and one part of disorder omitted, only one of the three identical subunits shown for clarity) Selected bond lengths (Å) and angles (deg.) for **9**: Mn1–Cl1 = 2.3737(5); Mn1–Cl2A = 2.3549(18); Mn1–Cl3A = 2.3728(10); Mn1–Cl4A = 2.4025(15); N1–O1 = 1.1922(17); N6–O6 = 1.191(2); Cl1–Mn1–Cl2A = 108.62(6); Cl3A–Mn1–Cl4A = 103.78(6).

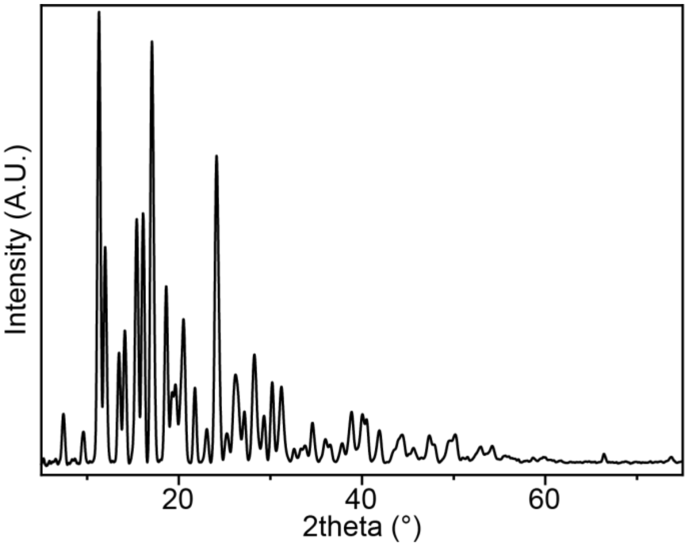

Figure S 18:PXRD spectra of **9**.

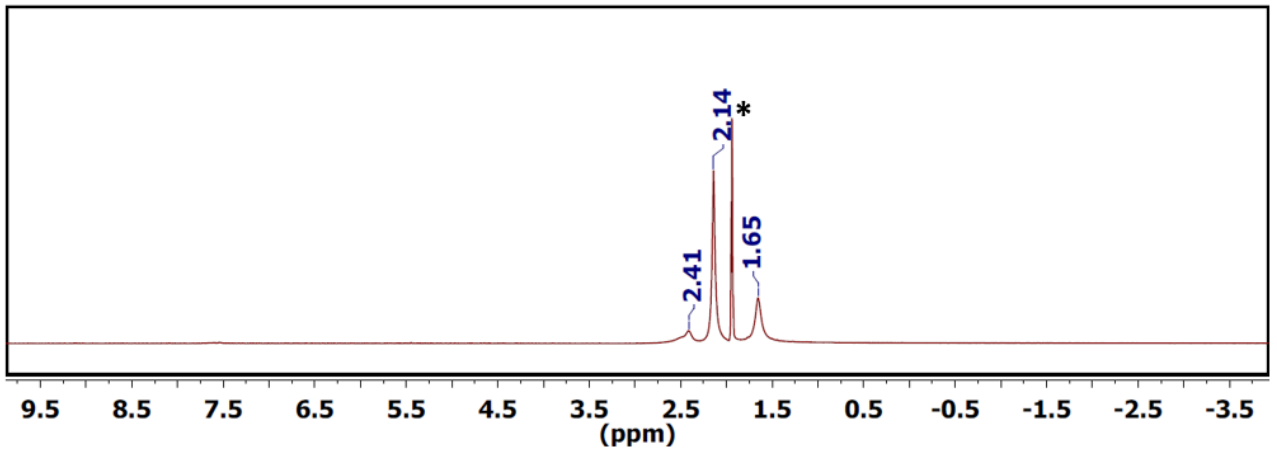

Figure S19: ^{1}H-NMR spectrum of [TEMPO]BF_4 in CD_3CN(*).

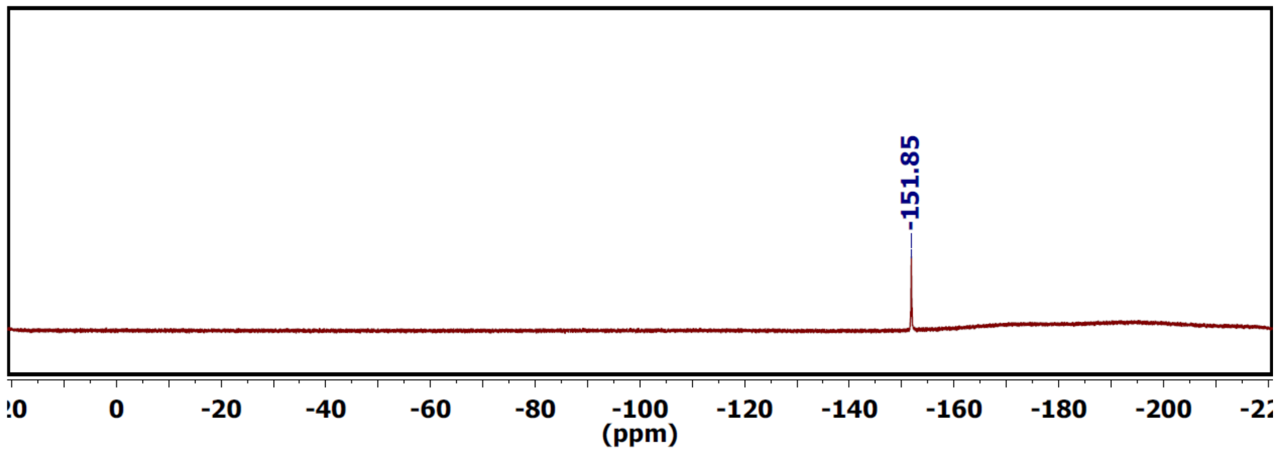

Figure S20: ^{19}F-NMR spectrum of [TEMPO]BF_4 in CD_3CN.

Electrochemistry experiments

Complex	Potential (V) vs. $FeCp_2$	
	From CV	From DPV
$[MnCl(H_2O)(PyNO)_4][MnCl_4]$ (**5**)	0.488	0.460
$[Mn(PyNO)_4Cl_2]_2[MnCl_4]$•$[Mn(PyNO)_2Cl_3]$ (**6•7**)	0.448, 0.869	0.471, 0.910
$[MnCl_3(PyNO)_2]$ (7)	0.472	0.471

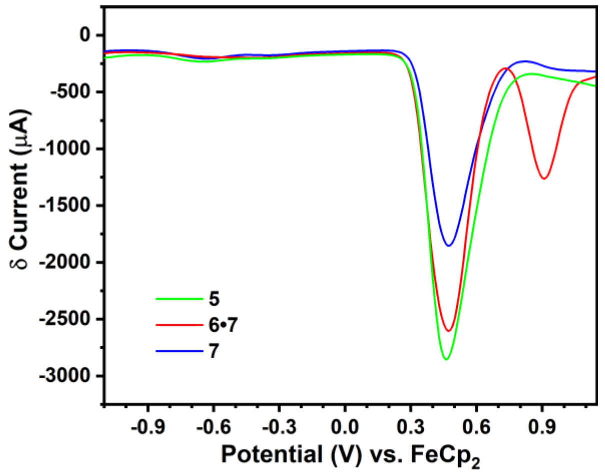

Figure S21: Overlaid differential pulse voltammograms of **5**,**6•7**, and **7** in 0.5 M [$n$$Bu_4N$][$PF_6$] in MeCN.

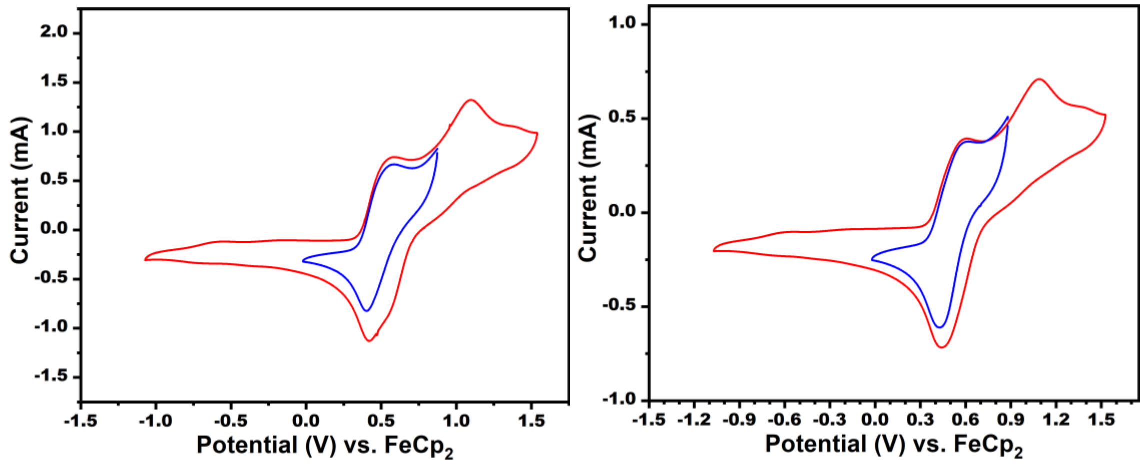

Figure S22: (Left) The cyclic voltammogram of **5** (red) in 0.5 M [nBu$_4$N][PF$_6$] in MeCN overlaid with the Mn^{III}/Mn^{II} reversible event (blue). (Right) The cyclic voltammogram of **7** (red) in 0.5 M [nBu$_4$N][PF$_6$] in MeCN overlaid with the Mn^{III}/Mn^{II} reversible event (blue).

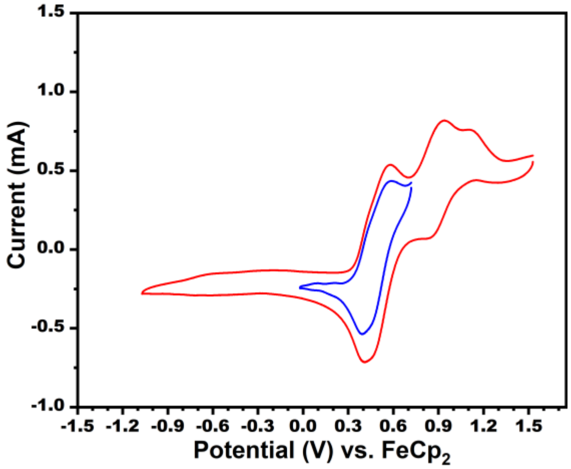

Figure S23: (Left) The cyclic voltammogram of **6•7** (red) in 0.5 M [nBu$_4$N][PF$_6$] in MeCN overlaid with the Mn^{III}/Mn^{II} reversible event(blue).

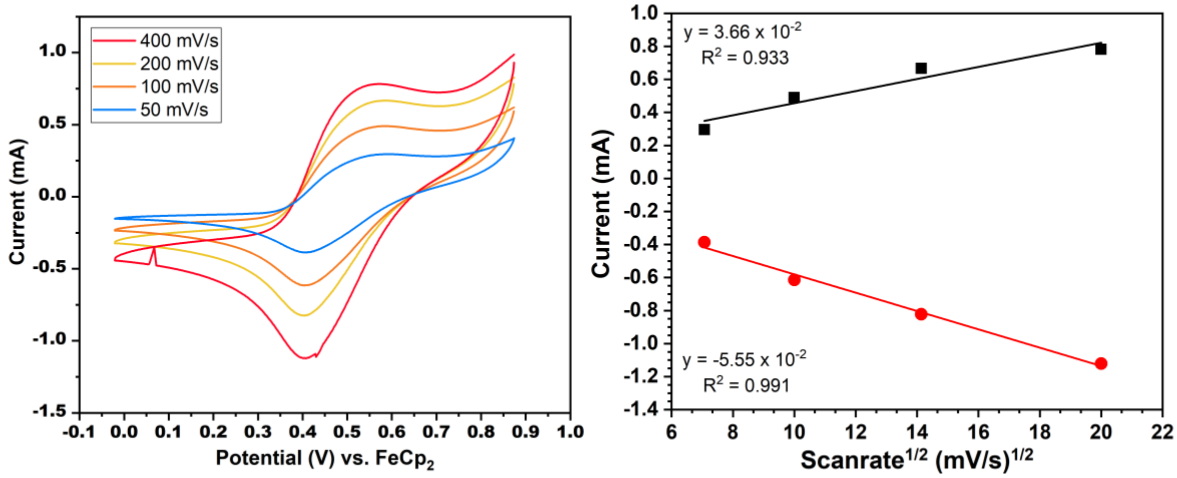

Figure S24: (Left) Cyclic voltammograms of **5** in 0.5 M [nBu$_4$N][PF$_6$] in MeCN at varying scan rates. (Right) Peak current vs. (scan rate)$^{1/2}$ with linear fit.

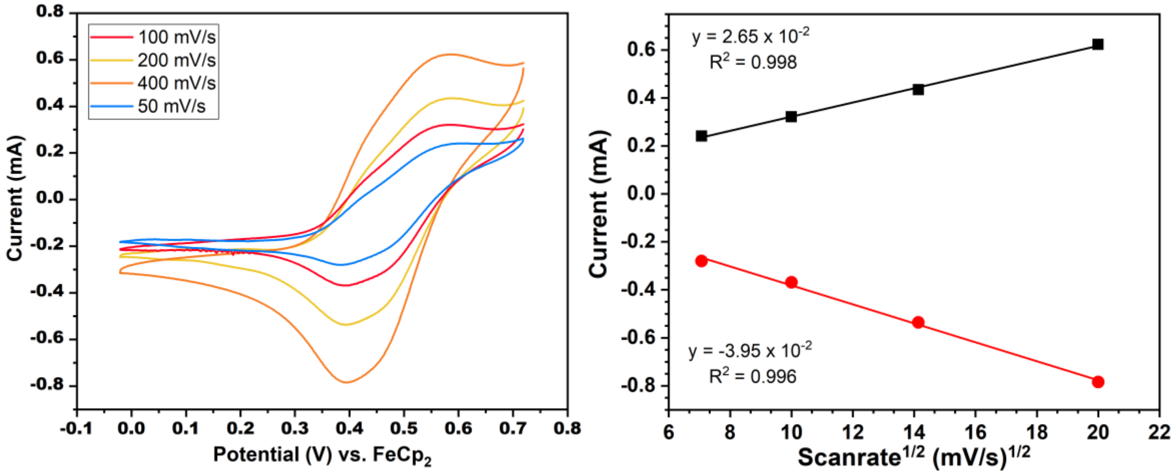

Figure S25: (Left) Cyclic voltammograms of **6•7** in 0.5 M [*n*Bu$_4$N][PF$_6$] in MeCN at varying scan rates. (Right) Peak current vs. (scan rate)$^{1/2}$ with linear fit.

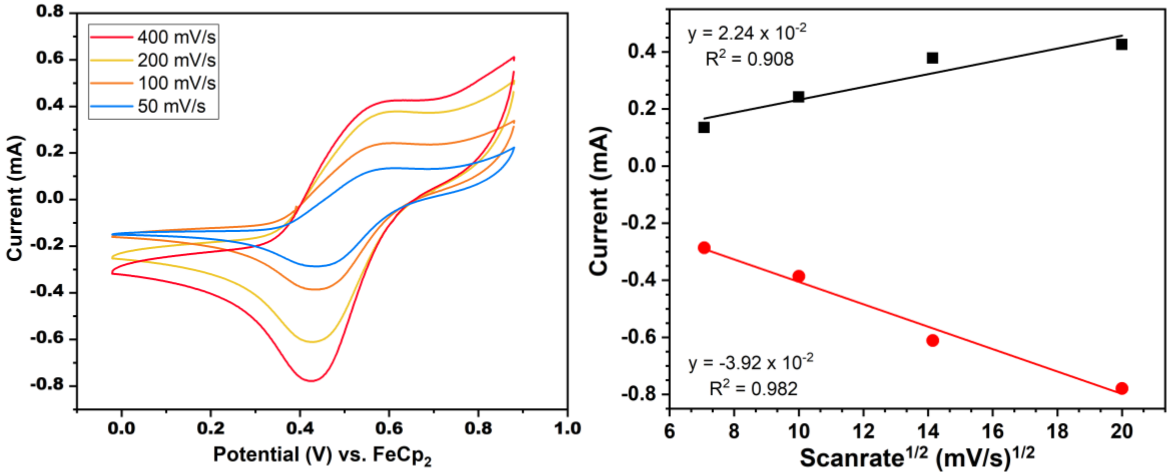

Figure S26: (Left) Cyclic voltammograms of **7** in 0.5 M [*n*Bu$_4$N][PF$_6$] in MeCN at varying scan rates. (Right) Peak current vs. (scan rate)$^{1/2}$ with linear fit.
